# Efficacy of A Virtual Agent–Based Digital Intervention (Echo App V2.0) for Patients With Alcohol Use Disorder: Randomized Controlled Trial

**DOI:** 10.2196/91065

**Published:** 2026-09-17

**Authors:** Shuo Li, Dapeng Zhang, Weicong Sang, Ruihua Li, Wei Yuan, Jingyang Liu, Huixue Li, Meimei Liu, Yunchun Zhang, Bingqian Zhang, Qianying Wu, Chuanning Huang, Haidi Shan, Jiang Du, Haifeng Jiang, Na Zhong, Tianzhen Chen, Min Zhao

**Affiliations:** 1 Shanghai Mental Health Center, Shanghai Jiao Tong University School of Medicine and School of Psychology, No. 600 of Wanping Road, Xuhui District, Shanghai, 200300, China, 86 18017311005; 2 Fuyang No. 3 People's Hospital, Fuyang, China; 3 Shandong Mental Health Center, Jinan, Shandong, China; 4 Peking University Sixth Hospital, Beijing, China; 5 GOS-UCL Institute of Child Health, London, United Kingdom

**Keywords:** conversational agent, digital intervention, virtual digital human, alcohol use disorder, randomized controlled trial

## Abstract

**Background:**

Virtual agents powered by dialogue trees have emerged as promising tools to overcome limitations of text-only psychological interventions in mental health treatment.

**Objective:**

This study evaluated whether a virtual agent–based digital psychological intervention plus treatment as usual (TAU) reduced craving and relapse and improved emotional state, sleep quality, and treatment motivation compared with TAU alone in patients with alcohol use disorder (AUD).

**Methods:**

A 2-arm, randomized controlled trial with a parallel-group design was conducted at Fuyang No. 3 People’s Hospital between April and December 2023. A total of 93 patients aged 18 to 55 years with AUD were randomly assigned to the intervention (n=47) or control group (n=46) using a random number table. Outcome assessors and data analysts were blinded to group allocation. Patients in the intervention group received the 4-week Echo App V2.0 intervention, comprising 10 cognitive behavioral therapy sessions (30‐45 min each). All patients received TAU, including medications, daily 30-minute exercise, and psychological education. The primary outcome was change in craving from T0 (baseline) to T1 (week 4), T2 (1 mo after discharge), and T3 (3 mo after discharge), measured by the Visual Analog Scale, a 10-cm horizontal line. Secondary outcomes were relapse and changes in emotional state, sleep quality, and treatment motivation.

**Results:**

Craving increased after discharge in both groups. The intervention group showed significantly attenuated craving increases at T2 (β=−1.81, 95% CI −3.45 to −0.16; *P*=.03) and T3 (β=−1.97, 95% CI −3.61 to −0.32; *P*=.02). At T2, the intervention group showed significantly lower relapse rates (χ^2^_1_=4.09; *P*=.04), although this benefit was sensitive to missing-data assumptions in sensitivity analyses. Relapse rates did not differ at T3 (χ^2^_1_=0.029, *P*=.86). Sleep quality improved more in the intervention group at T1 (β=−3.28, 95% CI −5.08 to −1.49; *P*<.001). Perceived stress decreased significantly over time (β=−2.91, 95% CI −5.27 to −0.56; *P*=.02). Group and change in Perceived Stress Scale (PSS) significantly predicted craving change at T2 (intervention: β=−2.13, 95% CI −4.03 to −0.24, *P*=.03; PSS: β=0.15, 95% CI 0.03 to 0.26; *P*=.02) and T3 (intervention: β=−2.24, 95% CI −4.07 to −0.42, *P*=.02; PSS: β=0.12, 95% CI 0.01 to 0.24, *P*=.03). Both group (β=1.02, odds ratio 2.77, 95% CI 1.11 to 6.91; *P*=.03) and number of previous attempts to quit alcohol (β=0.27, odds ratio 1.31, 95% CI 1.06 to 1.63; *P*=.01) significantly predicted remission at T2. No adverse events were reported.

**Conclusions:**

Echo App V2.0 attenuated postdischarge craving increases and may reduce short-term relapse among patients with AUD. However, the relapse finding was sensitive to missing-data assumptions and was not sustained at 3 months. Postdischarge continuation strategies may be needed to maintain benefits.

## Introduction

Alcohol use disorder (AUD) is a problematic pattern of alcohol use that leads to significant impairment or distress and contributes to mortality globally, causing 3 million deaths per year [1-3]. Severe AUD, the most serious form of problematic alcohol consumption, is easy to become chronic and need long-term medical and psychological management [4]. In China, the overall prevalence of AUD reached 3.2%, with the rate among males reaching 10.1%. Despite the high prevalence of AUD, only approximately one-sixth of individuals with AUD receive treatment, with lower treatment rates in underdeveloped regions [5].

Currently, treatment for AUD is still limited [6]. The imbalance between professionals and people in need causes low accessibility of psychotherapy, with merely 0.219 psychotherapists per 10,000 persons in China [7]. Previous studies also found that low treatment rates are related to personal financial circumstances, stigma, attitudes and beliefs, as well as the cost of treatment and lack of professionals [8-13].

In the past years, digital medicine has developed rapidly and provides effective and cost-efficient treatment [2,14-16]. Digital interventions could enhance treatment flexibility and accessibility, promote patient-centered care, foster a sense of empowerment and autonomy, and improve adherence to standardized treatment [17,18]. Previous digital interventions primarily delivered services to patients through text, animations, or conversational interfaces. However, these interventions have problems such as poor interactive experience and high dropout rates. Virtual agents (VAs), with vivid human-like images and the ability to interact with users, have the potential to significantly enhance trustworthiness, intervention effectiveness, and patient adherence [19,20]. However, few studies are based on VAs for mental disorders, especially on how to improve the effectiveness of treatment as usual (TAU). The evidence base for digital psychotherapeutic interventions in AUD has advanced considerably in recent years. A web-based cognitive behavioral therapy (CBT) program (CBT4CBT) produced greater increases in alcohol abstinence over 8 months compared with both clinician-delivered CBT and standard outpatient care, with patients assigned to digital CBT improving their percentage of days abstinent by more than 50% [21]. A 12-week mobile CBT app yielded outcomes comparable to face-to-face CBT for outpatients with alcohol use problems [22]. However, reviews have consistently identified engagement and dropout as major limitations of text-based or minimally interactive platforms, underscoring the need for more immersive delivery formats [17]. VA-based systems with three-dimensional embodiment, voice synthesis, and empathetic responsiveness represent a qualitative advance over text-only tools. Therefore, we developed a VA-based digital intervention called the Echo app, which functions as a virtual digital psychotherapist. Preliminary results of the pilot study on individuals with methamphetamine use disorders have been promising. Participants reported a significant improvement in their motivation to abstain from drugs and overall satisfaction with the app [23]. Qualitative feedback from that study also informed revisions to session content and interaction flow for V2.0, including adding physiological monitoring, richer multimedia content (videos, animations), and more emotionally responsive, empathetic interactions [24]. Based on the pilot study and evidence-based psychological interventions, we developed Echo App V2.0 for AUD.

The Echo App V2.0 operates through a semistructured human-computer interaction. This system integrates advanced technologies, including a comprehensive AI-driven animation solution, 3D modeling, binding technologies, speech synthesis, and intelligent interaction capabilities, to create the VA image. In contrast to traditional text-based self-help agents, this system fosters a much more immersive and interactive experience. Echo App V2.0 uses inquiries to guide the treatment process, steer the conversation, and collect participant information by posing questions. Following the participant’s responses, the system provides targeted feedback in the form of statements and recommendation summaries. To reinforce therapeutic outcomes, the system includes knowledge assessments that test the participant’s understanding of key psychotherapeutic concepts using multiple-choice or true/false questions. Moreover, prior digital interventions have primarily targeted broad mechanisms such as general psychoeducation and basic emotional support, without systematically addressing the full range of psychotherapeutic processes relevant to AUD [25]. In contrast, Echo App V2.0 was designed as a structured, session-based virtual psychotherapist specifically targeting evidence-based mechanisms underlying AUD recovery: psychoeducation on alcohol and craving, CBT-based cognitive restructuring, motivational enhancement, emotion regulation, stress management, sleep quality improvement, and relapse prevention [21,26-30]. Stress is recognized as a key contributor to alcohol misuse, as individuals often use alcohol to cope with psychological distress [31]. Given this relationship, addressing stress alongside drinking behaviors may enhance intervention efficacy. These therapeutic targets were operationalized across 10 structured sessions (Table S1 in Multimedia Appendix 1).

**Figure 1.**
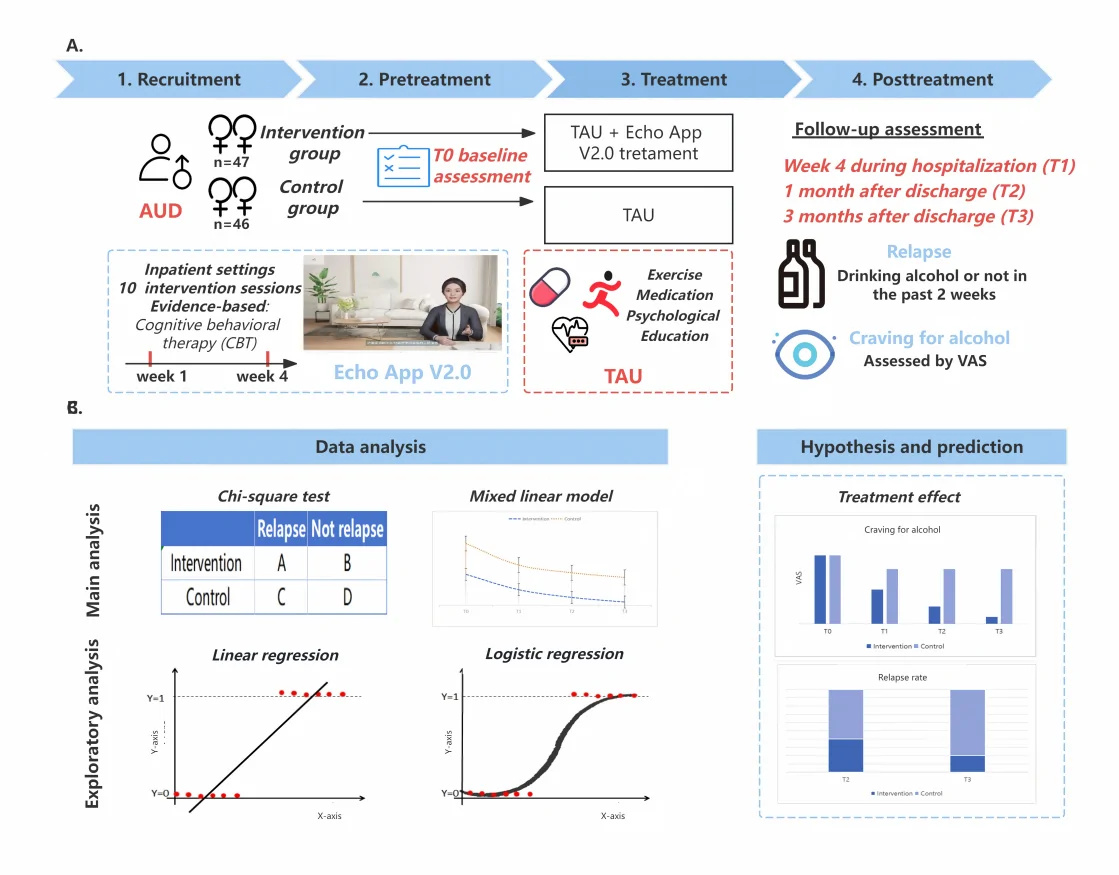
Study abstract. A 2-arm, single-blind randomized controlled trial conducted at Fuyang No. 3 People’s Hospital, China (April-December 2023), comparing a virtual agent–based digital psychological intervention (Echo App V2.0) combined with treatment as usual (TAU) vs TAU alone in 93 hospitalized adults aged 18-55 years with diagnosed alcohol use disorder. The abstract depicts study design, intervention components, primary outcome (alcohol craving via Visual Analog Scale [VAS]), and secondary outcomes (relapse, sleep quality, emotional state, and treatment motivation) assessed at baseline (T0), week 4 (T1), 1 month (T2), and 3 months after discharge (T3).

Specifically, this study aimed to assess whether the combination of Echo App V2.0 and TAU yields greater efficacy compared with TAU alone [32]. We hypothesized that the intervention group would demonstrate a significantly greater attenuation of alcohol craving over time and lower relapse rates. Furthermore, we hypothesized that the VA intervention would lead to greater improvements in secondary clinical indicators, including emotional state, sleep quality, and perceived stress. The abstract of the study is shown in Figure 1.

## Methods

### Trial Design

This study employed a 2-arm, single-blind randomized controlled trial (RCT) with a parallel-group design and a 1:1 randomization ratio. Participants were randomly allocated to 2 treatment conditions. The intervention group received Echo App V2.0 treatment in addition to TAU, while the control group received only TAU. Both groups were assessed at treatment initiation (T0) and week 4 (T1), as well as 1 month (T2) and 3 months (T3) after they were discharged from the hospital. All patients in this study were discharged from the hospital within 10 days after treatment. The study protocol was approved by the institutional review board of Shanghai Mental Health Center (REC:2020‐92C2).

### Patient and Public Involvement

Participants were recruited from Fuyang No. 3 People’s Hospital between April and December 2023 through advertisements. The first participant was enrolled on April 15, 2023. Inclusion criteria were age 18 to 55 years, meeting *DSM-5* (*Diagnostic and Statistical Manual of Mental Disorders, Fifth Edition*) AUD criteria, alcohol abstinence for <3 months [33], computer literacy, and informed consent. Exclusion criteria were comorbid neuropsychiatric diseases, cognitive impairment, a family history of mental illness, and inability to complete assessments.

### Sample Size

Drawing from prior literature on innovative interventions for individuals with AUD [9], to achieve 80% statistical power to detect a medium effect size (Cohen *d*=0.56) in the trajectory of craving scores between the intervention and control groups, assuming 4 measurement time points with the correlation of 0.6 between repeated measures, a total of 35 participants were required for each group. In anticipation of a 30% dropout rate, a total sample size of 100 participants was required, with 50 participants per group.

### Randomization and Blinding

A total of 93 participants were randomly assigned (1:1) to the intervention (Echo App V2.0 + TAU, n=47) or control (TAU, n=46) groups using a random number table. The random allocation sequence was generated by an independent statistician using a computer-generated random number table prior to trial commencement. Participants were enrolled by treating clinicians. Group assignment was performed by a designated research coordinator who was not involved in participant recruitment or outcome assessment. Allocation concealment was maintained using sequentially numbered, opaque, sealed envelopes, which were opened only after a participant had provided informed consent and been formally enrolled. Given the nature of the intervention, neither participants nor treating clinicians could be blinded to group assignment. To mitigate expectation effects, both groups received equivalent clinician contact time. Control participants received standard psychological education as part of TAU, ensuring that the between-group comparison reflected the specific contribution of the Echo App V2.0 beyond general attention and psychoeducation. Researchers responsible for outcome assessment and data analysis were blinded to group allocation throughout the study period.

### Intervention and Comparator

#### VA-Based Digital Intervention Delivered by the Echo App V2.0

The intervention in this study was the VA-based intervention delivered through the Echo App V2.0. The intervention was developed to improve the accessibility of comprehensive rehabilitation for individuals with AUD by providing standardized, evidence-based psychological treatment through a virtual psychotherapist. Its therapeutic content was informed by 3 evidence-based psychological approaches recommended for AUD treatment: motivational interviewing, CBT, and mindfulness-based interventions (MBIs).

Participants were recruited from Fuyang Third People’s Hospital, and the intervention was delivered exclusively on hospital-provided tablet computers during the inpatient admission. The intervention consisted of 10 CBT sessions (30‐45 min each) delivered 2 to 3 times weekly over 4 weeks during hospitalization. Before treatment, participants completed a multidimensional assessment, and assessment results were automatically integrated by the system to generate individualized treatment recommendations, after which the virtual therapist delivered the treatment sessions and provided automated feedback throughout the intervention.

Treatment sessions covered treatment motivation, impulsivity, coping strategies, stress, anxiety, depression, sleep quality, and compulsion through semistructured human-computer interaction with a virtual psychotherapist avatar and audio-guided mindfulness exercises. The virtual character was designed as a 3D-modeled guide to enhance engagement and orientation within the app. Navigation followed a structured pathway: core modules were preplanned to ensure clinical fidelity. Session completion and engagement data were recorded automatically by the app server. Adherence was defined as completing at least 8 of the 10 prescribed sessions. Fidelity was monitored weekly by a supervising clinician who reviewed session logs; technical failures or incomplete sessions were flagged and, where possible, rescheduled within the 4-week inpatient window. The Echo App V2.0 was deployed exclusively on hospital-provided tablets and was accessible only during the inpatient period.

#### Technical Architecture and Therapeutic Content Basis of the Echo App V2.0

The Echo App V2.0 operates on a rule-based, script-driven dialogue architecture. All utterances delivered by the VA are preauthored text strings rendered at runtime via speech-synthesis technology. The app runs in server mode: the user-side tablet communicates with a central server that stores assessment data and manages session logic across the patient, administrator, and server tiers [32]. The VA does not query an external knowledge base at runtime. The therapeutic content of the VA was analogous to the training curriculum of a human practitioner and was developed by a multidisciplinary team of psychiatrists and clinical psychologists at Shanghai Mental Health Center and was grounded in 3 evidence-based psychological frameworks recommended for AUD: motivational interviewing, CBT, and MBIs. Specific source materials drawn upon included: (1) the CBT model for substance use disorders as described by Carroll and Kiluk [30], covering cognitive restructuring, high-risk situation identification, and relapse prevention; (2) the motivational interviewing principles of Miller and Rollnick [34], operationalized to enhance abstinence motivation through decisional-balance exercises; (3) mindfulness techniques for relapse prevention, operationalized in stress management and sitting/mountain meditation; and (4) psychoeducational content on alcohol pharmacology, craving mechanisms, emotion regulation, sleep hygiene, and well-being. Scripts were reviewed and iteratively revised by the clinical team to ensure fidelity to the source CBT frameworks before deployment. The specific themes and content of these 10 sessions are provided in Table S1 in Multimedia Appendix 1.

#### TAU

Standardized 4-week hospitalization including medications (benzodiazepines for withdrawal symptoms, tapered over 7‐10 d), daily 30-minute exercise (15 min Baduanjin, 15 min aerobic), and psychological education [35-38]. TAU components were standardized and applied uniformly to all participants in both arms throughout the 4-week inpatient period. No additional cointerventions were permitted or recorded in either group. Medication dosing was individually titrated by treating physicians according to withdrawal severity, but the protocol type remain ed consistent across participants.

### Outcomes

#### Primary Outcome

The primary outcome was alcohol craving measured by the Visual Analog Scale (VAS) across 4 time points. A 10 cm horizontal line was presented to participants, from the left end point labeled as “no craving at all” (0) to the right end point labeled as “extreme craving” (10). A change of 1.0 point or greater on the VAS has been proposed as a clinically meaningful difference in craving intensity in substance use populations [39]. The primary hypothesis concerned whether the trajectory of craving over time differed between the intervention and control groups.

#### Secondary Outcomes

The secondary outcome was alcohol relapse at follow-up assessment after participants were discharged from the hospital. Alcohol relapse was defined as whether the participants drank 4 or more (female) or 5 or more (male) standard drinks in the last 14 days [40]. We also defined patients lost to follow-up as having relapsed because previous studies have shown that when patients relapse, they usually avoid contact with therapists or researchers due to a sense of shame or frustration [41]. This conservative, intention-to-treat (ITT)-consistent approach is commonly used in substance use trials to avoid overestimating abstinence rates. To evaluate the robustness of this primary analysis, sensitivity analyses were conducted using alternative definitions: (1) respondents-only analysis using the standard clinical definition (participants lost to follow-up were excluded), and (2) best-case and worst-case scenario analyses (in the best-case scenario, all missing in the treatment group=nonrelapse and all missing in the control group=relapse; in worst-case scenario, the reverse).

Other measurements included treatment motivation evaluated with the Stages of Change Readiness and Treatment Eagerness Scale (SOCRATES), anxiety measured via the Generalized Anxiety Disorder-7 (GAD-7), depression evaluated by the Patient Health Questionnaire-9 (PHQ-9), stress gauged through the Perceived Stress Scale (PSS), and sleep quality assessed by the Pittsburgh Sleep Quality Index (PSQI). The Chinese versions of the scales have good reliability and validity. The Chinese versions of all secondary outcome scales have been validated in Chinese populations and demonstrate good psychometric properties: GAD-7 (Cronbach α=0.90) [42], PHQ-9 (α=0.86) [43], PSQI (α=0.73) [44], PSS (α=0.85) [45], and SOCRATES [46]. No prespecified clinical thresholds were applied to secondary outcomes, and all were analyzed as continuous variables. Coping style and impulsivity were prespecified secondary outcomes, assessed using the Simplified Coping Style Questionnaire and Barratt Impulsiveness Scale, respectively. As these outcomes are beyond the primary scope of the present report, their detailed results are provided in Table S5 in Multimedia Appendix 1.

### Adverse Events and Safety

Adverse events and participant safety were monitored throughout the study. No serious adverse events related to the intervention were recorded. Participants showing signs of clinical deterioration were referred to the clinical team for evaluation. No participants were withdrawn from the study due to intervention-related harm. Digital interactions were reviewed weekly by the clinical supervisor to identify any unexpected distress.

### Statistical Methods

R software (version 4.4.2; R Foundation for Statistical Computing) was used with a 2-sided α=.05 and 95% CI. Baseline variables were summarized by mean (SD) or frequency (%). Independent *t* tests were applied to normally distributed data. All analyses followed the ITT principle. Generalized linear mixed models (GLMMs) assessed changes in VAS and secondary outcomes (PSQI, GAD-7, PHQ-9, PSS, SOCRATES), with fixed effects for group, time, and group × time. For the primary analysis of the craving outcome, GLMMs were used with missing data imputed using last observation carried forward (LOCF). For the relapse outcome, missing data were handled by classifying all participants with missing outcome data as having relapsed. Chi-square analysis was conducted to evaluate the response of relapse or remission.

For sensitivity analyses, 2 approaches were employed to assess the robustness of findings. For alcohol craving, missing data were addressed using MI, with 50 imputation datasets generated using predictive mean matching and pooled according to Rubin’s rules [47]. A tipping-point analysis was further conducted to nullify the observed treatment effect, thereby evaluating the robustness of the craving findings [48]. For relapse data, we first calculated the respondents-only situation (missing=missing: participants lost to follow-up were not included in the analysis). Then, best-case and worst-case scenario analyses were performed: in the best-case scenario, all missing values in the intervention group were imputed as nonrelapse and all missing values in the control group as relapse; the worst-case scenario applied the reverse assumption [49].

We conducted a linear regression model to explore the influencing factors for craving. Variables such as intervention assignment and alcohol use history were also selected. Additionally, all other potential variables were analyzed individually to preliminarily identify those significantly associated with craving for alcohol at follow-up. Variables with a *P* value less than .2 were selected as candidate variables for inclusion in the multivariable model. Finally, the selected variables were entered into stepwise regression to determine the final model. We conducted a logistic model to explore the influencing factors for relapse or remission using the same variable selection procedure.

### Ethical Considerations

The study protocol was approved by the institutional review board of Shanghai Mental Health Center (REC: 2020‐92C2). Written informed consent was obtained from all participants prior to enrollment, clearly explaining the study’s purpose, procedures, and their right to withdraw at any time without affecting their standard medical care. To protect privacy and confidentiality, all collected study data were strictly deidentified and stored on secure, encrypted servers accessible only to the core research team. Participants received RMB 20 (US $2.98; CNY ¥1=US $0.149 as of September 5, 2026) in financial compensation for completing the follow-up assessments. Additionally, no identifiable images or personal details of individual participants are presented in this manuscript or its supplementary materials.

### Protocol Deviations

Alcohol relapse was elevated to a key secondary outcome postregistration due to its clinical relevance in AUD postdischarge care. Secondary measures (Barratt Impulsiveness Scale and Simplified Coping Style Questionnaire) were moved to the Supplementary Appendix to maintain manuscript focus. T3 reflects the 3-month postinpatient follow-up window specified in the protocol.

## Results

### Sample Characteristics

**Figure 2.**
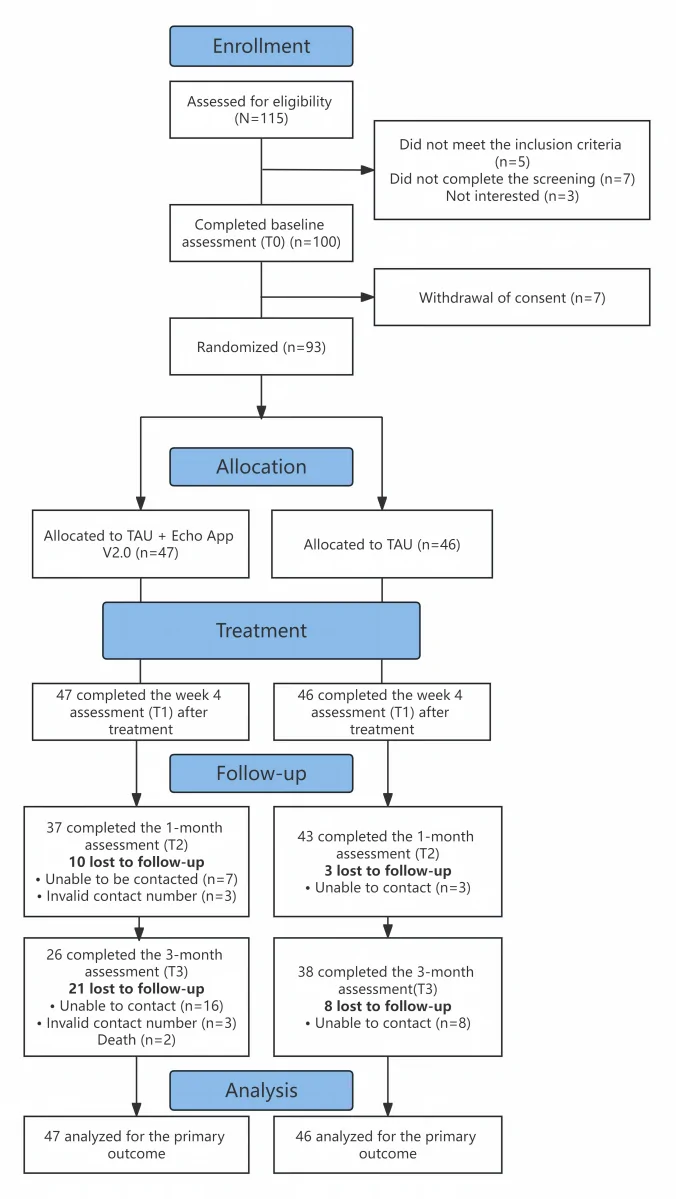
CONSORT (Consolidated Standards of Reporting Trials) flow diagram of participant enrollment, allocation, follow-up, and analysis. Of 115 adults screened, 93 eligible participants aged 18-55 years with *DSM-5* (*Diagnostic and Statistical Manual of Mental Disorders, Fifth Edition*) alcohol use disorder were randomized (1:1) to the intervention group (n=47) or control group (n=46). Follow-up assessments were conducted at week 4 (T1), 1 month (T2), and 3 months (T3). Dropout rates were 14% at T2 and 31% at T3. All 93 participants were included in intention-to-treat analyses.

Fifteen of 115 participants assessed for eligibility were excluded. Of these, 7 subsequently withdrew consent, and the remaining 93 participants were randomized to the intervention group (TAU + Echo App V2.0, n=47) or the control group (TAU only, n=46). Thirteen participants and 29 participants dropped out at T2 (dropout rate: 14%) and T3 (dropout rate: 31%), respectively. With regard to intervention fidelity, 83.0% (39/47) of participants in the intervention group completed all 10 sessions; the mean number of sessions completed was 9.2 (SD 1.3). Six participants completed fewer than 8 sessions, most commonly due to early discharge (n=4) or device technical issues (n=2). No major protocol deviations were recorded. A total of 93 participants were included in ITT analyses. The CONSORT (Consolidated Standards of Reporting Trials) flow diagram is shown in Figure 2 [50,51] (Checklist 1).

The average age of the participants was 39.06 (SD 8.37) years, with an average of 9.03 (SD 3.57) years of education. Income was evenly distributed, ranging from less than 3000 yuan (US $447.01; CNY ¥1=US $0.1490 as of September 5, 2026) to over 10,000 yuan (US $1490.02). The average duration of alcohol use was 19.83 years, with an average of 2.38 attempts at quitting alcohol (Table 1).

**Table 1.** Baseline sociodemographic and clinical characteristics^a^.

Characteristic	Total population	Intervention group (n=47)	Control group (n=46)	*t* test (91)/Chi-square (*df*)	*P* value
Age (y), mean (SD)	39.06 (8.37)	38.36 (8.37)	39.78 (8.40)	0.817	.42
Marital status, n (%)				χ² (2)=4.948	.08
Married	58 (62.4)	33 (70.2)	25 (54.3)		
Single	12 (12.9)	7 (14.9)	5 (10.9)		
Divorced	23 (24.7)	7 (14.9)	16 (34.8)		
Years of education, mean (SD)	9.03 (3.57)	8.85 (3.67)	9.22 (3.50)	0.493	.62
Employment status, n (%)				χ² (1)=0.048	.83
Employed	76 (81.7)	38 (80.9)	38 (82.6)		
Unemployed	17 (18.3)	9 (19.1)	8 (17.4)		
Income (yuan)^b^, n (%)				χ² (3)=3.129	.37
<3000	22 (23.7)	10 (21.3)	12 (26.1)		
3000‐5000	30 (32.3)	15 (31.9)	15 (32.6)		
5000‐10000	27 (29.0)	17 (36.2)	10 (21.7)		
More than 10,000	14 (15.1)	5 (10.6)	9 (19.6)		
Place of residence, n (%)				χ² (1)=0.860	.35
Urban	42 (45.16)	19 (40.4)	23 (50)		
Towns and rural areas	51 (54.84)	28 (59.6%)	23 (50)		
Duration of alcohol use (years), mean (SD)	19.83 (9.91)	18.89 (9.87)	20.80 (9.97)	0.922	.36
Frequency of alcohol consumption, n (%)				χ² (2)=0.670	.72
Once per week	20 (21.5)	10 (21.3)	10 (21.7)		
Three to five times per week	27 (29.0)	12 (25.5)	15 (32.6)		
Daily	46 (49.5)	25 (53.2)	21 (45.7)		
Attempts to quit alcohol, n	2.38	2.31	2.91	1.294	.20

a Intervention group (Echo App V2.0 + TAU, n=47); control group (TAU, n=46). Data presented as mean (SD) or frequency (%). No significant between-group differences were observed at baseline.

b CNY ¥1=US $0.1490 as of September 5, 2026.

### Treatment Effects

#### Craving for Alcohol

**Figure 3.**
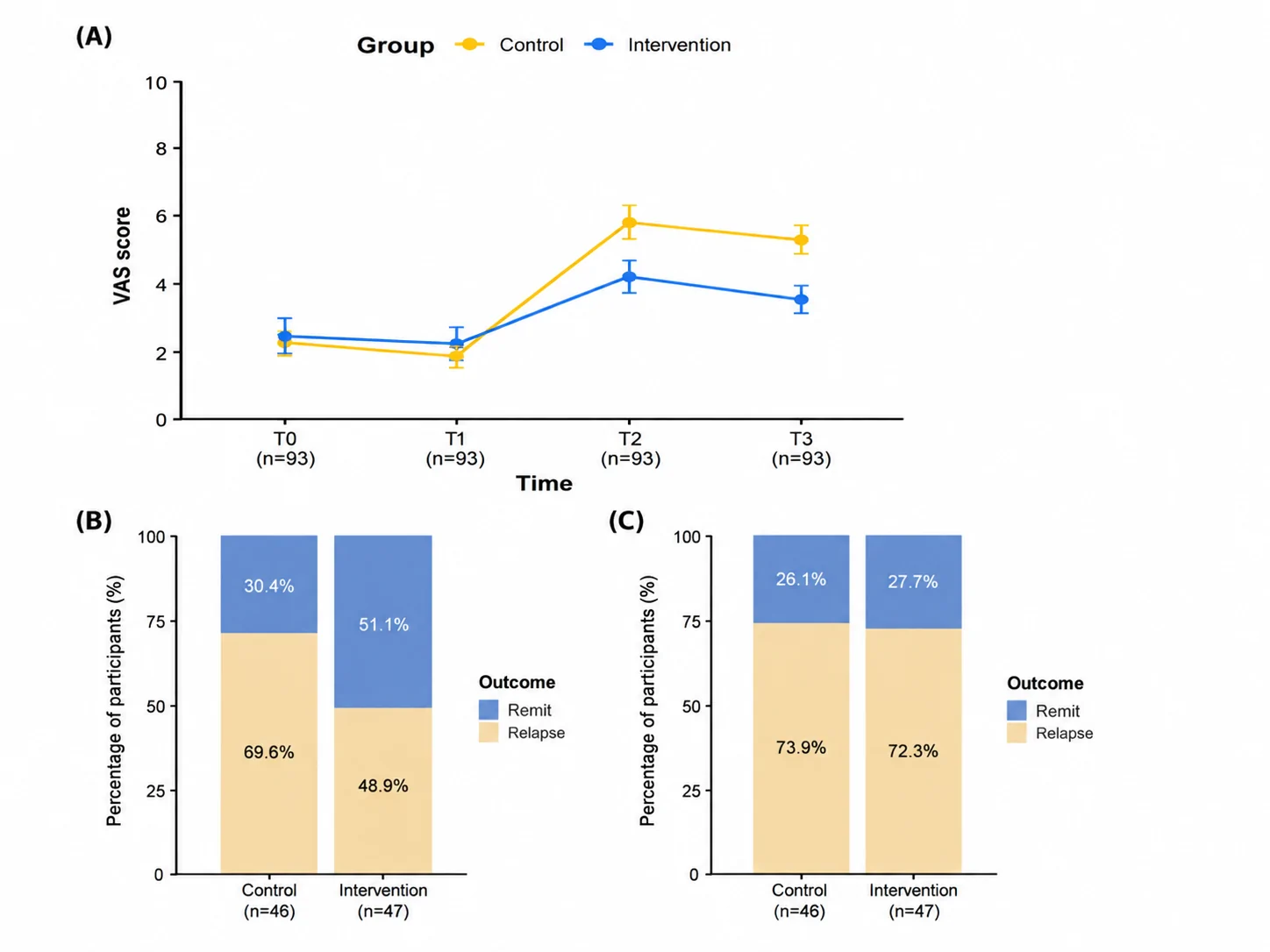
Primary outcome measures. (A) Mean alcohol craving scores (VAS, 0‐10) by group over 4 time points. Significant group × time interactions at T2 (β=−1.81, *P*=.03) and T3 (β=−1.97, *P*=.02); (B) alcohol relapse at 1-month follow-up (T2): intervention 48.9% vs control 69.6% (χ^2^_1_=4.09, *P*=.04); (C) alcohol relapse at 3-month follow-up (T3): 72.3% vs 73.9% (nonsignificant). Intervention group: virtual agent–based digital intervention (Echo App V2.0) combined with treatment as usual; control group: treatment as usual. T0: baseline; T1: week 4 follow-up; T2: 1-month follow-up; T3: 3-month follow-up. VAS: Visual Analog Scale.

Both groups showed an increase in craving after discharge. Figure 3 illustrates that the time effect on craving scores was significant between T0 and T2 (β=5.349, 95% CI 2.732 to 7.967; *P*<.001; Cohen *d*=1.885) and T3 (β=5.002, 95% CI 2.518 to 7.486; *P*<.001; Cohen *d*=1.763). However, the effect at T1 was not significant (β=−0.546, 95% CI −3.167 to 2.074; *P*=.68; Cohen *d*=−0.193). The effect of the intervention was not significant (β=0.225, 95% CI −0.973 to 1.423; *P*=.71; Cohen *d*=0.079). The interaction effect between time and group was significant at T2 (β=−1.805, 95% CI −3.445 to −0.164; *P*=.03; Cohen *d*=−0.636) and T3 (β=−1.965, 95% CI −3.606 to −0.324; *P*=.02; Cohen *d*=−0.693). Specifically, the intervention group showed a significantly attenuated craving increase at T2 and T3. Table S3 in Multimedia Appendix 1 provides the absolute estimated levels at each time point for direct comparison.

To assess the robustness of the primary craving outcome, MI was performed. Pooled GLMM results were consistent with the primary analysis in both direction and magnitude (Table S4 in Multimedia Appendix 1). Tipping-point analysis further confirmed the robustness of the craving findings. For VAS at T2, 60.2% (1012/1681) of all tested missingness scenarios yielded significant results, with a minimum perturbation of +2.5 points in the intervention group required to nullify significance. For VAS at T3, 72.9% (1225/1681) of scenarios remained significant, requiring a combined perturbation of 5.0 points across both groups to reverse the conclusion. These findings suggest that the treatment effect on alcohol craving is robust to plausible departures from the missing-at-random (MAR) assumption.

#### Other Measurements

In the primary analysis (N=93, missing=relapse), the relapse rates at T2 and T3 were 59.14% and 73.12%, respectively. At T2, 55 participants relapsed, while 38 achieved remission. In the intervention group, 23 participants relapsed and 24 remitted (relapse rate: 48.9%). In the control group, 32 participants relapsed and 14 remitted (relapse rate: 69.6%).

The chi-square analysis found a significant difference between the 2 groups (Figure 3, χ^2^_1_=4.094, *P*=.04). At T3, 68 participants relapsed and 25 remitted. In the intervention group, 34 participants relapsed and 13 remitted (relapse rate: 72.3%). In the control group, 34 participants relapsed and 12 remitted (relapse rate: 73.9%). The chi-square analysis indicated no significant difference (Figure 3C, χ^2^_1_=0.029, *P*=.86).

In the respondents-only sensitivity analysis, in which we only count the standard clinical definition among respondents as relapse (N=93), the chi-square results indicated that the intervention group showed a significantly lower relapse rate at T2 compared with the control group (intervention: 35.1% vs control: 67.4%; χ^2^_1_=7.08, *P*=.008). At T3, the difference was not statistically significant (intervention: 50.0% vs control: 68.4%; χ^2^_1_=2.20, *P*=.14). Best-case and worst-case scenario analyses demonstrated the range of possible findings. In the worst-case scenario (N=93, control: missing=missing, intervention: missing=relapse), the T2 difference was nonsignificant (intervention: 48.9% vs control: 63.0%; χ^2^_1_=1.88, *P*=.17), as was the T3 difference (intervention: 72.3% vs control: 56.5%; χ^2^_1_=2.54, *P*=.11). In the best-case scenario (N=93, control: missng=relapse, intervention: missig=missing), both T2 (intervention: 27.7% vs control: 69.6%; χ^2^_1_=16.35, *P*<.001) and T3 (intervention: 27.7% vs control: 73.9%; χ^2^_1_=19.90, *P*<.001) showed highly significant differences.

**Figure 4.**
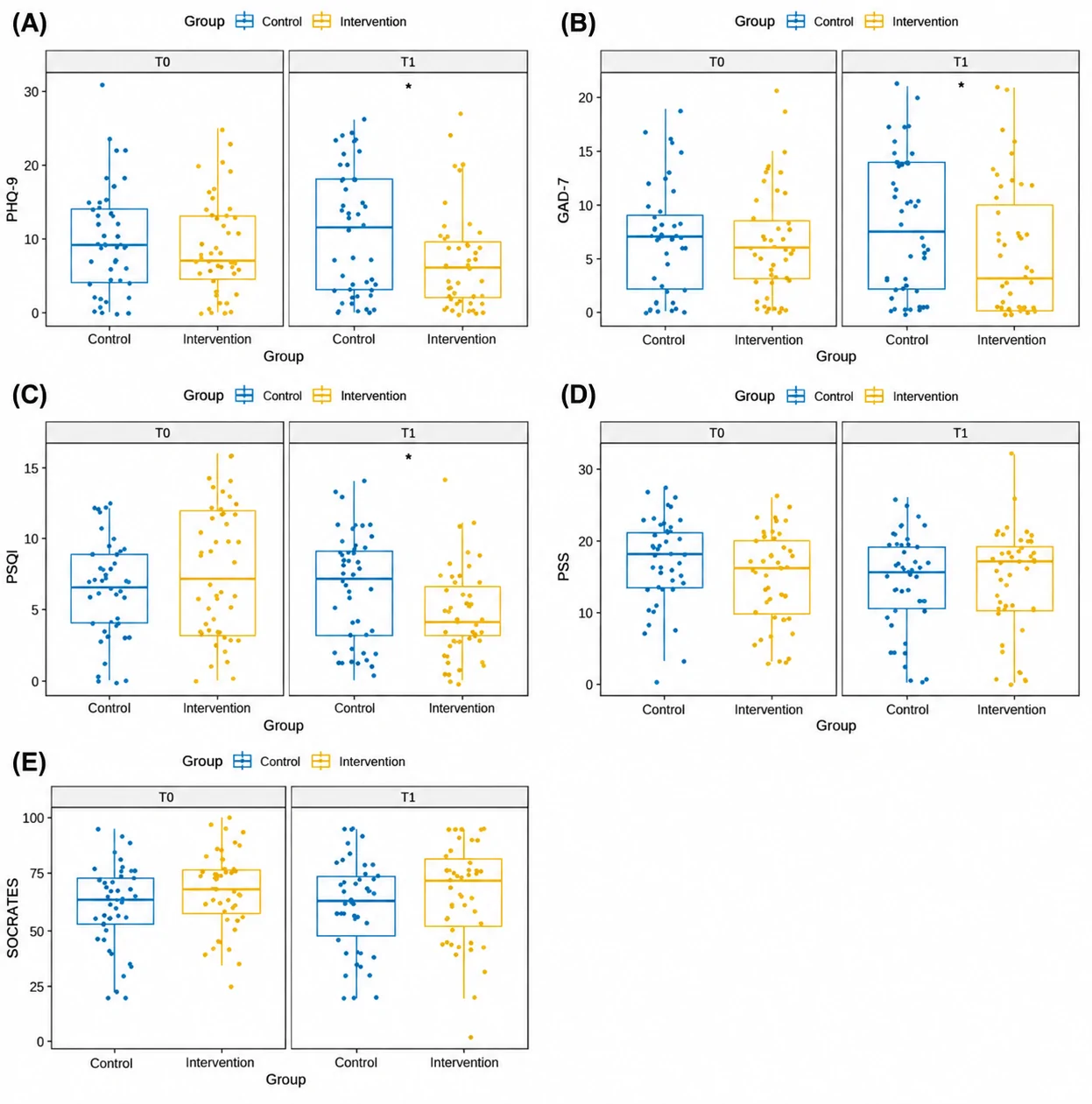
Secondary outcome measures: the only significant group × time interaction was PSQI sleep quality (β=−3.28, *P*<.001 at T1); no significant between-group differences were observed for PHQ-9, GAD-7, PSS, or SOCRATES. (A) PHQ-9 change over time; (B) GAD-7 change over time; (C) PSQI change over time; (D) PSS change over time; (E) SOCRATES change over time. Intervention group: VA-based digital intervention (Echo App V2.0) combined with treatment as usual; control group: treatment as usual. GAD-7: Generalized Anxiety Disorder-7; PHQ-9: Patient Health Questionnaire-9; PSQI: Pittsburgh Sleep Quality Index; PSS: Perceived Stress Scale; SOCRATES: Stages of Change Readiness and Treatment Eagerness Scale; T0: baseline; T1: week 4 follow-up; T2: 1-month follow-up; T3: 3-month follow-up.

The results of the GLMM analysis for other secondary indicators are in presented Table S2 in Multimedia Appendix 1 and Figure 4. For PHQ-9, the fixed effect of time (β=0.870, 95% CI −1.282 to 3.022; *P*=.42; Cohen *d*=0.169) and intervention was nonsignificant (β=−1.197, 95% CI −4.213 to 1.819; *P*=.43; Cohen *d*=0.233). The interaction between time and intervention was not significant (β=−2.657, 95% CI −5.725 to 0.412; *P*=.08; Cohen *d*=0.517). There were no significant main effects of time (β=1.196, 95% CI −0.475 to 2.868; *P*=.15; Cohen *d*=0.305) or group (β=−0.471, 95% CI −2.848 to 1.906; *P*=.70; Cohen *d*=0.120) on GAD. The interaction between time and intervention was not significant (β=−1.877, 95% CI −4.171 to 0.417; *P*=.11; Cohen *d*=0.478). The fixed effect of time on PSQI scores was not statistically significant (β=0.304, 95% CI −0.973 to 1.582; *P*=.64; Cohen *d*=0.099). The effect of intervention showed nonsignificant (β=1.330, 95% CI −0.150 to 2.810; *P*=.10; Cohen *d*=0.432). The interaction effect was statistically significant (β=−3.283, 95% CI −5.080 to −1.485; *P*<.001; Cohen *d*=1.066). For the PSS scores, the main effect of time was significant (β=−2.913, 95% CI −5.267 to −0.559; *P*=.02; Cohen *d*=0.516). The effect of the intervention is nonsignificant (β=−2.424, 95% CI −5.301 to 0.453; *P*=.09; Cohen *d*=0.429). However, the interaction effect between time and group was not significant (β=2.253, 95% CI −1.091 to 5.597; *P*=.18; Cohen *d*=0.399).

The fixed effect of time on SOCRATES scores was not significant (β=0.065, 95% CI −5.587 to 5.718; *P*=.98; Cohen *d*=0.005). The effect of intervention showed nonsignificant (β=6.550, 95% CI −1.666 to 14.766; *P*=.12; Cohen *d*=0.483). Additionally, the interaction effect was nonsignificant (β=−0.661, 95% CI −8.598 to 7.275; *P*=.87; Cohen *d*=0.049).

#### Ancillary Analyses

**Figure 5.**
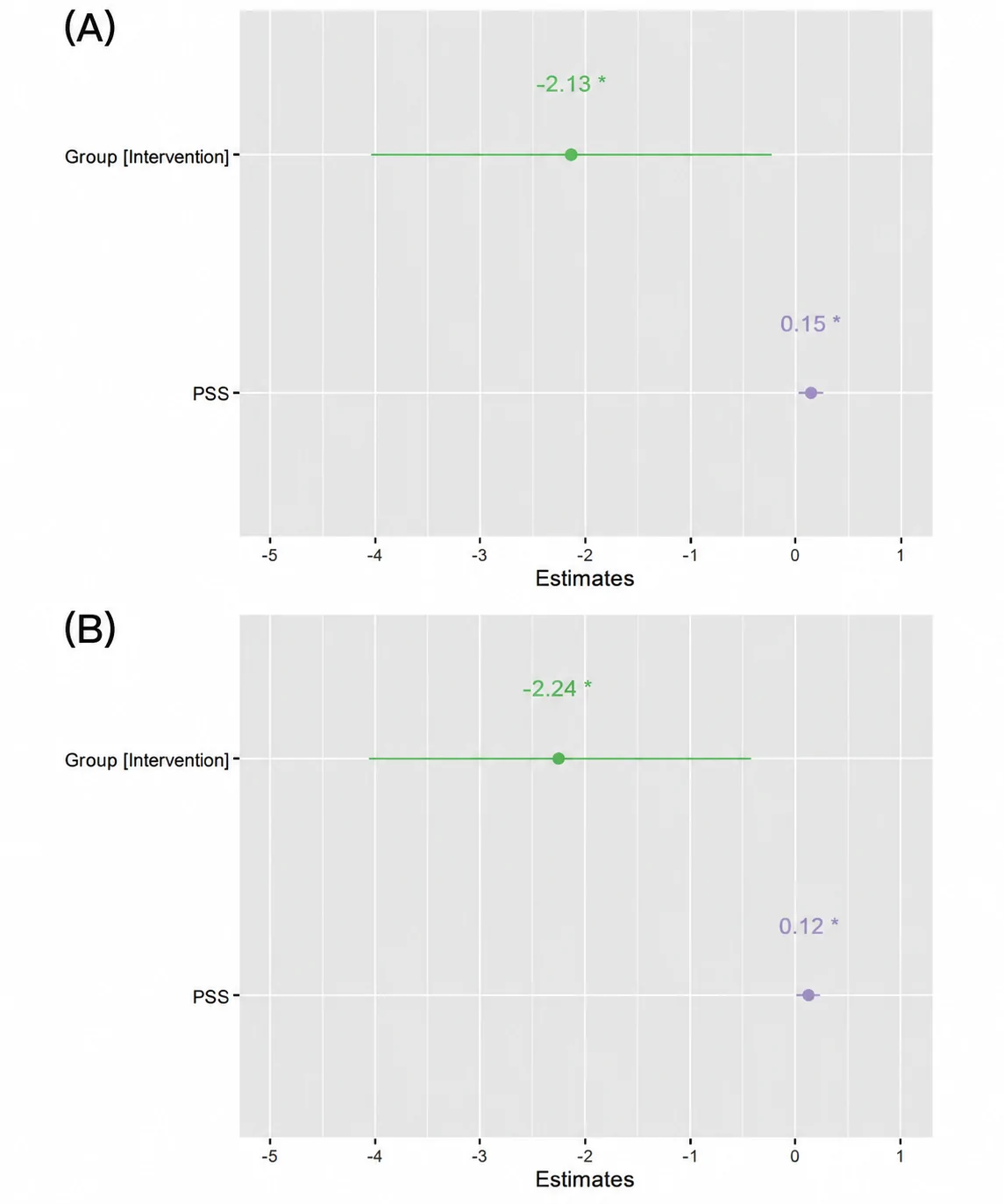
Linear regression. (A) ΔVAS T0-T2: group (β=−2.13, *P*=.03) and PSS (β=0.15, *P*=.02) were significant predictors (*R*^2^=0.097). (B) ΔVAS T0-T3: group (β=−2.24, *P*=.02) and PSS (*β*=.12, *P*=.03; *R*²=0.094). PSS: Perceived Stress Scale; T0: baseline; T1: week 4 follow-up; T2: 1-month follow-up; T3: 3-month follow-up; VAS: Visual Analog Scale.

For the difference between VAS scores at T2 and T0 (Figure 5), the overall model was statistically significant (*F*_2,90_=4.841, *P*=.01), with an *R*^2^ value of 0.097. After adjusting for the predictors, the adjusted *R*^2^ was 0.077. The intercept of the model was 3.40 (95% CI 2.07 to 4.72, *P*<.001). There was a significant negative effect of intervention on the VAS change (β=−2.133, 95% CI −4.032 to −0.235; *P*=.03). Additionally, higher PSS scores were significantly associated with a greater increase in craving change (β=0.146, 95% CI 0.028 to 0.263; *P*=.02). Variance inflation factors (VIF=1.02) indicated no multicollinearity between predictors. The Breusch-Pagan test (BP=3.82, *P*=.15) confirmed homoscedasticity of residuals, and the Shapiro-Wilk test (*W*=0.979, *P*=.14) verified that residuals were normally distributed. Ten-fold cross-validation yielded an *R*² of 0.138, exceeding the in-sample *R*² of 0.097, indicating that the model did not overfit the data.

For the difference between craving scores at T3 and T0 (Figure 5), the overall model was statistically significant (*F*_2,90_=4.677, *P*=.01, *R*^2^=0.094). After adjusting for the predictors, the adjusted *R*^2^ was 0.074. There was a significant negative effect of intervention on the craving change (β=−2.243, 95% CI −4.067 to −0.419; *P*=.02). Higher PSS scores were significantly associated with a greater increase in craving change (β=0.123, 95% CI 0.010 to 0.236; *P*=.03). Variance inflation factors (VIF=1.02) indicated no multicollinearity between predictors. Ten-fold cross-validation yielded an *R*² of 0.162, exceeding the in-sample *R*² of 0.094, demonstrating satisfactory generalizability. Although the Shapiro-Wilk test indicated mild nonnormality of residuals (*W*=0.961, *P*=.007), bootstrap CIs (1000 resamples) were additionally computed to ensure inferential robustness. Bootstrap results were consistent with those obtained from parametric testing, confirming that the regression coefficients and their significance were not meaningfully affected by the departure from normality.

**Figure 6.**
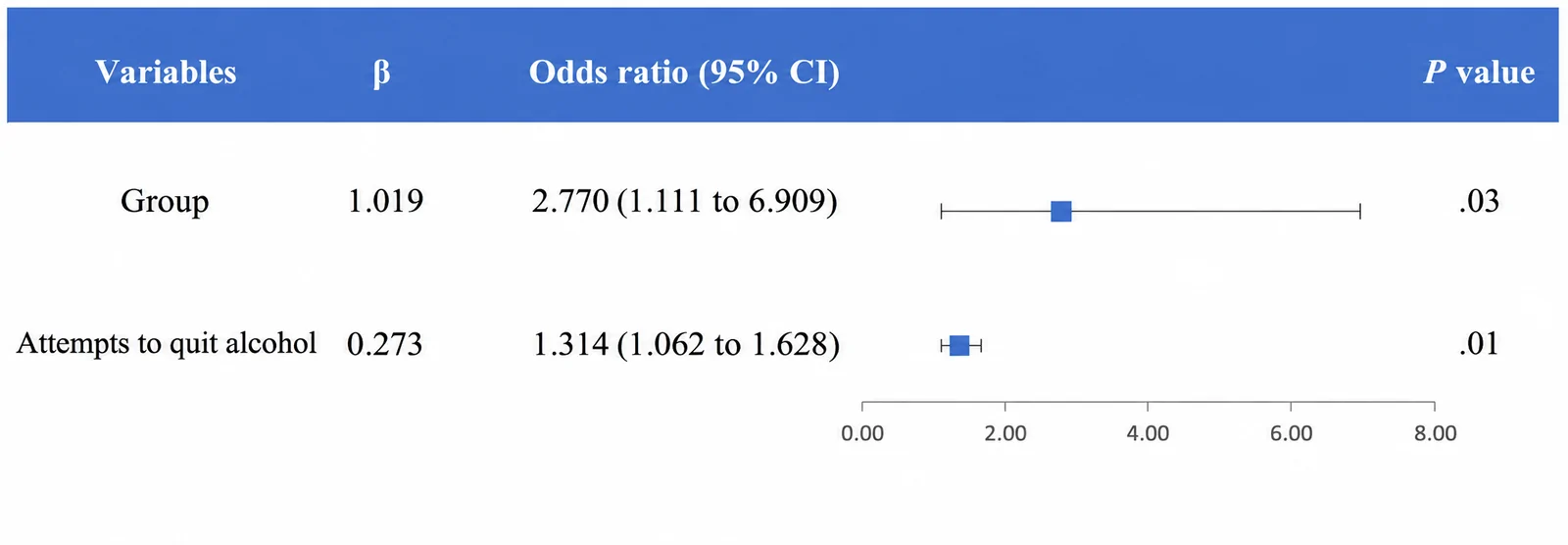
Logistic regression. Odds ratio (OR) >1 indicates higher odds of remission (nonrelapse) in the intervention group relative to the control group. Intervention assignment was associated with higher odds of remission (OR 2.77, 95% CI 1.11‐6.91; *P*=.03); attempts to quit alcohol predicted higher odds of remission (OR 1.31, 95% CI 1.06‐1.63; *P*=.01).

The logistic regression analysis used relapse at T2 as the dependent variable (Figure 6). The intervention group had significantly higher odds of remission compared with the control group (β=1.019, OR 2.770, 95% CI 1.111 to 6.909; *P*=.03). Additionally, the number of previous attempts to quit alcohol was positively associated with the odds of remission (β=0.273, OR 1.314, 95% CI 1.062 to 1.628; *P*=.01).

## Discussion

### Principal Findings

This study is, to our knowledge, among the first RCTs with mid-term follow-up (up to 3 months postdischarge) to test the effectiveness of a VA-based digital intervention in AUD during hospitalization. The intervention group demonstrated a significantly attenuated increase in craving at 1 month and 3 months postdischarge. An early reduction in relapse was also observed at 1 month, although this finding should be interpreted cautiously because it was sensitive to assumptions regarding missing outcome data. Importantly, no between-group difference in relapse was evident at 3 months, suggesting that the intervention’s benefit on relapse was not sustained after discharge. Additionally, the intervention group experienced greater improvements in sleep quality. However, the between-group difference in relapse rates was no longer significant at the 3-month mid-term follow-up. Linear regression analysis also suggested that stress can effectively predict changes in craving at 2 follow-ups postdischarge. The logistic regression showed that both the intervention and the number of previous attempts to quit alcohol can predict relapse.

Baseline craving scores were relatively low in both groups, reflecting the suppression of craving during inpatient detoxification. This may introduce floor effects that limit the detectable range of between-group differences during hospitalization. The postdischarge assessments therefore represent the primary window of clinical interest, when craving resurges following discharge. Alcohol craving in both groups remained low during hospitalization but experienced an increase followed by a decrease after discharge. A longitudinal study tracking hospitalized patients with AUD during their hospitalization also found that approximately half of the patients maintained relatively low levels of alcohol craving during hospitalization [52]. Despite gradual reduction in withdrawal symptoms over time, craving increased with the length of abstinence, peaking around 60 days of abstinence [53]. Another reason could be real-life stress and triggers after discharge [54]. This craving suppression likely contributed to the short-term advantage in relapse observed at the 1-month follow-up. The attenuated but persistent increase in craving across both groups at 3 months is consistent with the observed convergence of relapse rates between groups at that time point. Plenty of studies have clarified the association between alcohol craving and relapse risk [55]. Specifically, their increasing craving further exaggerated their reuse of alcohol [10,56].

Significant differences in alcohol relapse were observed between the 2 groups at 1 month. Both the intervention and the number of previous attempts to quit alcohol were significant predictors. However, the benefit on relapse was not sustained at 3 months, possibly due to disengagement and lack of continued intervention access, and further refinements are needed to sustain efficacy over time. Patients in the intervention group benefited from novelty and frequent support at first, but this effect diminished over time as they stopped using the digital intervention [17,57] and disengaged from the supportive environment of the hospital. Future iterations of the intervention should explore postdischarge access models to sustain therapeutic gains over time.

The validity of these findings must be considered in the context of postdischarge missing data, which in AUD trials is likely to be informative rather than purely random, given that patients who relapse tend to disengage from follow-up. The primary analysis employed LOCF imputation. To evaluate the robustness of the primary findings under potential departures from MAR, we conducted multiple sensitivity analyses using alternative missing data assumptions. In the sensitivity analyses, MI with 50 datasets was performed under the MAR assumption using full information maximum likelihood. For alcohol craving, the pooled GLMM results from MI were consistent with the primary LOCF-based analysis in both direction and magnitude, supporting the reliability of the craving outcomes. Tipping-point analysis further indicated that the craving effects at both 1 month and 3 months were resistant to plausible levels of missingness-related bias, requiring substantial hypothetical perturbations to nullify significance.

For relapse outcomes, treating missing data as missing, both best- and worst-case scenario analyses indicated that the 1-month benefit was generally robust to different missing data assumptions, with statistical significance lost only under the most conservative (worst-case) scenario. At 3 months, however, a significant difference was observed only under the most optimistic (best-case) assumption, suggesting that the intervention effect on relapse was not sustained over time. These sensitivity analyses support the robustness of the primary findings: while the craving outcomes remain stable across assumptions, the 1-month relapse benefit is somewhat sensitive to extreme assumptions about missing data, and the lack of a sustained effect at 3 months is unlikely to be driven by missingness alone. Overall, the intervention provides encouraging evidence of early clinical benefit, whereas evidence for sustained relapse prevention beyond 1 month remains insufficient.

Echo App V2.0 also significantly improved participants’ sleep quality. Sleep disorders are common comorbidities of AUD and potential pathogenic factors for relapse [58-62]. Furthermore, sleep disturbances are commonly observed following alcohol withdrawal or detoxification [63]. Previous research also found that among patients with AUD, poorer sleep quality is associated with higher levels of depression, worse quality of life, and psychological distress [64,65]. Improving sleep quality can serve as a complementary therapy for AUD. A meta-analysis also indicated that insomnia interventions can improve sleep quality and alleviate depressive symptoms in patients with AUD [66].

Perceived stress improved over time in both groups, without a significant between-group difference, suggesting that this reduction most likely reflects a general benefit of inpatient abstinence rather than a specific effect of the VA intervention. This is consistent with the well-established relationship between alcohol removal and stress relief [67,68]. Stress-related mechanisms contribute significantly to craving and relapse susceptibility. Past research has revealed statistically significant correlations between social adversity, childhood and adult trauma, stressful events, and the risk of addiction [69]. Neuroimaging research has also revealed that stress increases cravings for alcohol by activating the brain’s stress response system, such as the hypothalamic-pituitary-adrenal axis [70]. Alcohol, as a short-term stress reliever, can inhibit the activity of the hypothalamic-pituitary-adrenal axis and relieve stress.

Linear regression analysis also suggested that stress can effectively predict changes in craving. Patients with high stress levels are more likely to have higher cravings for alcohol. To achieve better effectiveness, interventions for AUD need to target stress disturbances in addiction [31]. Echo App V2.0 has a session for stress management and incorporates mindfulness intervention. A systematic review has shown that MBIs for substance misuse yield large effects in reducing stress [71]. An implication of this finding is that digital interventions for AUD need to integrate effective treatments for stress, especially mindfulness, and care for patients with high stress levels. The logistic regression showed that both the intervention and number of previous attempts to quit alcohol can predict relapse. Repeated attempts to quit alcohol imply that the patients might have undergone recurrent setbacks and failures during the course of these attempts [72]. This could reflect the insufficiency of the patient’s coping mechanisms for alcohol abstinence. The more attempts to quit drinking, the more pronounced the flaws in the patient’s coping mechanisms might be, thereby escalating the risk of relapse. We acknowledge that stepwise regression with a liberal screening threshold may increase the risk of model instability and capitalize on chance associations. Results from the regression analyses should therefore be interpreted as exploratory rather than confirmatory, and replication in larger independent samples is warranted.

Digital interventions are very diverse in terms of course, target users, and techniques [17]. The meta-analysis of previous studies indicates significant reductions in alcohol consumption, frequency, and heavy episodic drinking after receiving digital interventions [73]. However, there still exist concerns about accessibility, privacy and safety, and engagement. Previous qualitative studies indicated that patients require support, supervision, monitoring, and follow-up from clinicians in order to effectively engage in digital interventions [74].

VA-based digital interventions have great potential in reducing alcohol use among the general population. VAs could achieve better user experience through empathy, nonverbal relational behaviors, and disclosure of personal information [75]. Digital interventions based on VAs have shown effectiveness in sleep regularity, depression prevention, distress and anxiety reduction, and adherence improvement [20,76-79].

### Limitations

Despite the potential of using VAs in treating AUD, there are several limitations. First, there is a lack of long-term follow-up for secondary measures such as emotions and sleep. The long-term effects of the intervention on emotions and sleep are still unclear. Future studies could further explore the underlying mechanisms by which the long-term follow-up and intervention effects diminish over time. Second, for craving, we used a subjective VAS questionnaire. Craving is inherently a subjective experience, but it can be assessed by cognitive paradigms such as the cue-exposure paradigm, which exposes individuals to cues associated with alcohol use while recording physiological changes associated with the urge to drink [80]. Third, all participants were recruited from a single inpatient facility, and findings may not extend to outpatient clinics, community settings, or primary care, where patients typically present with less severe AUD and different environmental triggers. The study’s age restriction precludes generalization to older adults or adolescents, who may differ in technology engagement and treatment needs. Multicenter, multisetting trials enrolling more diverse samples are needed before broad clinical implementation. Self-management after discharge has not been further clarified [81]. Further studies should validate the potential of Echo App V2.0 to provide self-management support for patients postdischarge. Specifically, future work should assess the feasibility of deploying Echo App V2.0 on patients’ own devices following discharge, evaluate the acceptability of a postdischarge “booster” session schedule, and examine whether continued app engagement mediates longer-term relapse outcomes. The postdischarge phase represents a critical period in AUD recovery during which patients face heightened exposure to environmental triggers, stress, and social pressure to drink. An app-delivered self-management tool that extends the therapeutic frameworks already introduced during hospitalization, such as CBT-based coping strategies and mindfulness techniques, could provide timely and accessible support precisely when it is most needed. Besides, more personalized treatment plans can be further implemented in the future.

### Conclusion

This RCT represents a methodological innovation as among the first studies with mid-term follow-up to rigorously test a 3D embodied VA-based digital psychotherapist for AUD during hospitalization. Distinct from prior text-based and minimally interactive digital interventions that suffer from high dropout rates, the Echo App V2.0 integrates advanced immersive technologies, including 3D avatar embodiment, speech synthesis, and empathic responsive interaction to deliver standardized, evidence-based therapeutic content.

The Echo App V2.0 has demonstrated certain therapeutic potential in treating AUD, especially craving, alcohol relapse, sleep quality, and psychological distress. Although an early reduction in relapse was observed at 1 month, this finding depended on assumptions regarding missing data and was not sustained at 3 months. These findings carry broader implications for how VA-assisted interventions might help address the persistent gap between the need for and the provision of evidence-based psychological treatment for AUD, particularly in settings with limited access to trained clinicians. Future development should therefore prioritize continuity of care models, including postdischarge booster sessions, app-based self-management tools, and adaptive delivery to improve durability of treatment effects. VA-based interventions can cement their role as scalable, accessible, and evidence-supported tools for bridging the global treatment gap in AUD care, particularly benefiting low-resource settings where the shortage of mental health professionals remains a critical barrier to treatment access.

## Supplementary material

10.2196/91065 Multimedia Appendix 1 Supplementary tables S1-S6 (detailed).

10.2196/91065 Checklist 1 CONSORT checklist.

## Abbreviations

AUD: alcohol use disorder
CBT: cognitive behavioral therapy
CONSORT: Consolidated Standards of Reporting Trials
*DSM-5*: *Diagnostic and Statistical Manual of Mental Disorders, Fifth Edition*
GAD-7: Generalized Anxiety Disorder-7
GLMM: generalized linear mixed model
ITT: intention-to-treat
LOCF: last observation carried forward
MAR: missing-at-random
MBI: mindfulness-based intervention
MI: multiple imputation
PHQ-9: Patient Health Questionnaire-9
PSQI: Pittsburgh Sleep Quality Index
PSS: Perceived Stress Scale
RCT: randomized controlled trial
SOCRATES: Stages of Change Readiness and Treatment Eagerness Scale
TAU: treatment as usual
VA: virtual agent
VAS: Visual Analog Scale
