# Supplementary material for: Efficacy of A Virtual Agent–Based Digital Intervention (Echo App V2.0) for Patients With Alcohol Use Disorder: Randomized Controlled Trial
Source: J Med Internet Res. 2026 Sep 17;28:e91065. doi: 10.2196/91065 (PMC13585020; doi:10.2196/91065)
Supplement: Supplementary file 1 [file jmir-v28-e91065-s002.pdf]

CONSORT-EHEALTH (V 1.6.1) - Submission/Publication Form

The CONSORT-EHEALTH checklist is intended for authors of randomized trials evaluating web-based and Internet-based applications/interventions, including mobile interventions, electronic games (incl multiplayer games), social media, certain telehealth applications, and other interactive and/or networked electronic applications. Some of the items (e.g. all subitems under item 5 - description of the intervention) may also be applicable for other study designs.

The goal of the CONSORT EHEALTH checklist and guideline is to be
a) a guide for reporting for authors of RCTs,
b) to form a basis for appraisal of an ehealth trial (in terms of validity)

CONSORT-EHEALTH items/subitems are MANDATORY reporting items for studies published in the Journal of Medical Internet Research and other journals / scientific societies endorsing the checklist.

Items numbered 1., 2., 3., 4a., 4b etc are original CONSORT or CONSORT-NPT (non-pharmacologic treatment) items.
Items with Roman numerals (i., ii, iii, iv etc.) are CONSORT-EHEALTH extensions/clarifications.

As the CONSORT-EHEALTH checklist is still considered in a formative stage, we would ask that you also RATE ON A SCALE OF 1-5 how important/useful you feel each item is FOR THE PURPOSE OF THE CHECKLIST and reporting guideline (optional).

Mandatory reporting items are marked with a red *.
In the textboxes, either copy & paste the relevant sections from your manuscript into this form - please include any quotes from your manuscript in QUOTATION MARKS,
or answer directly by providing additional information not in the manuscript, or elaborating on why the item was not relevant for this study.

YOUR ANSWERS WILL BE PUBLISHED AS A SUPPLEMENTARY FILE TO YOUR PUBLICATION IN JMIR AND ARE CONSIDERED PART OF YOUR PUBLICATION (IF ACCEPTED).
Please fill in these questions diligently. Information will not be copyedited, so please use proper spelling and grammar, use correct capitalization, and avoid abbreviations.

DO NOT FORGET TO SAVE AS PDF _AND_ CLICK THE SUBMIT BUTTON SO YOUR ANSWERS ARE IN OUR DATABASE !!!

Citation Suggestion (if you append the pdf as Appendix we suggest to cite this paper in the caption):
Eysenbach G, CONSORT-EHEALTH Group
CONSORT-EHEALTH: Improving and Standardizing Evaluation Reports of Web-based and Mobile Health Interventions
J Med Internet Res 2011;13(4):e126

URL: http://www.jmir.org/2011/4/e126/
doi: 10.2196/jmir.1923
PMID: 22209829

ls520522@gmail.com 切换账号

草稿未保存

未共享的内容

* 表示必填

Your name *

First Last

Shuo Li

Primary Affiliation (short), City, Country *

University of Toronto, Toronto, Canada

Shanghai Mental Health Center, Shanghai Jiao

Your e-mail address *

abc@gmail.com

ls520522@sjtu.edu.cn

Title of your manuscript *

Provide the (draft) title of your manuscript.

Efficacy of Virtual Agent-Based Digital Intervention (Echo-App V2.0) for Patients with Alcohol Use Disorder: A Randomised Controlled Trial

Name of your App/Software/Intervention *

If there is a short and a long/alternate name, write the short name first and add the long name in brackets.

Echo-App V2.0

Evaluated Version (if any)

e.g. "V1", "Release 2017-03-01", "Version 2.0.27913"

您的回答

Language(s) *

What language is the intervention/app in? If multiple languages are available, separate by comma (e.g. "English, French")

Chinese

URL of your Intervention Website or App

e.g. a direct link to the mobile app on app in appstore (itunes, Google Play), or URL of the website. If the intervention is a DVD or hardware, you can also link to an Amazon page.

您的回答

URL of an image/screenshot (optional)

您的回答

Accessibility *

Can an enduser access the intervention presently?

- ○ access is free and open
- ◉ access only for special usergroups, not open
- ○ access is open to everyone, but requires payment/subscription/in-app purchases
- ○ app/intervention no longer accessible
- ○ 其他:

Primary Medical Indication/Disease/Condition *

e.g. "Stress", "Diabetes", or define the target group in brackets after the condition, e.g. "Autism (Parents of children with)", "Alzheimers (Informal Caregivers of)"

Alcohol Use Disorder

Primary Outcomes measured in trial *

comma-separated list of primary outcomes reported in the trial

change of craving from T0(baseline) to T1(we

Secondary/other outcomes

Are there any other outcomes the intervention is expected to affect?

Secondary outcomes were relapse and change of emotional state, sleep quality, and treatment motivation

Recommended "Dose" *

What do the instructions for users say on how often the app should be used?

- ◯ Approximately Daily
- ◯ Approximately Weekly
- ◯ Approximately Monthly
- ◯ Approximately Yearly
- ◯ "as needed"
- ◉ 其他: 2-3 times weekly over 4 weeks

Approx. Percentage of Users (starters) still using the app as recommended after 3 months *

- ◯ unknown / not evaluated
- ◯ 0-10%
- ◯ 11-20%
- ◯ 21-30%
- ◯ 31-40%
- ◯ 41-50%
- ◯ 51-60%
- ◉ 61-70%
- ◯ 71%-80%
- ◯ 81-90%
- ◯ 91-100%
- ◯ 其他:

Overall, was the app/intervention effective? *

- [x] yes: all primary outcomes were significantly better in intervention group vs control
- [ ] partly: SOME primary outcomes were significantly better in intervention group vs control
- [ ] no statistically significant difference between control and intervention
- [ ] potentially harmful: control was significantly better than intervention in one or more outcomes
- [ ] inconclusive: more research is needed
- [ ] 其他:

Article Preparation Status/Stage *

At which stage in your article preparation are you currently (at the time you fill in this form)

- [ ] not submitted yet - in early draft status
- [ ] not submitted yet - in late draft status, just before submission
- [ ] submitted to a journal but not reviewed yet
- [x] submitted to a journal and after receiving initial reviewer comments
- [ ] submitted to a journal and accepted, but not published yet
- [ ] published
- [ ] 其他:

Journal *

If you already know where you will submit this paper (or if it is already submitted), please provide the journal name (if it is not JMIR, provide the journal name under "other")

- ○ not submitted yet / unclear where I will submit this
- ◉ Journal of Medical Internet Research (JMIR)
- ○ JMIR mHealth and UHealth
- ○ JMIR Serious Games
- ○ JMIR Mental Health
- ○ JMIR Public Health
- ○ JMIR Formative Research
- ○ Other JMIR sister journal
- ○ 其他:

Is this a full powered effectiveness trial or a pilot/feasibility trial? *

- ○ Pilot/feasibility
- ◉ Fully powered

Manuscript tracking number *

If this is a JMIR submission, please provide the manuscript tracking number under "other" (The ms tracking number can be found in the submission acknowledgement email, or when you login as author in JMIR. If the paper is already published in JMIR, then the ms tracking number is the four-digit number at the end of the DOI, to be found at the bottom of each published article in JMIR)

- ○ no ms number (yet) / not (yet) submitted to / published in JMIR
- ◉ 其他: #91065

TITLE AND ABSTRACT

1a) TITLE: Identification as a randomized trial in the title

1a) Does your paper address CONSORT item 1a? *

I.e does the title contain the phrase "Randomized Controlled Trial"? (if not, explain the reason under "other")

- (●) yes
- ( ) 其他:

1a-i) Identify the mode of delivery in the title

Identify the mode of delivery. Preferably use “web-based” and/or “mobile” and/or “electronic game” in the title. Avoid ambiguous terms like “online”, “virtual”, “interactive”. Use “Internet-based” only if Intervention includes non-web-based Internet components (e.g. email), use “computer-based” or “electronic” only if offline products are used. Use “virtual” only in the context of “virtual reality” (3-D worlds). Use “online” only in the context of “online support groups”. Complement or substitute product names with broader terms for the class of products (such as “mobile” or “smart phone” instead of “iphone”), especially if the application runs on different platforms.

	1	2	3	4	5	
subitem not at all important	○	○	○	○	●	essential

清除所选内容

Does your paper address subitem 1a-i? *

Copy and paste relevant sections from manuscript title (include quotes in quotation marks "like this" to indicate direct quotes from your manuscript), or elaborate on this item by providing additional information not in the ms, or briefly explain why the item is not applicable/relevant for your study

"Efficacy of Virtual Agent-Based Digital Intervention (Echo-App V2.0) for Patients with Alcohol Use Disorder: A Randomised Controlled Trial"

1a-ii) Non-web-based components or important co-interventions in title

Mention non-web-based components or important co-interventions in title, if any (e.g., “with telephone support”).

	1	2	3	4	5	
subitem not at all important	◉	○	○	○	○	essential

清除所选内容

Does your paper address subitem 1a-ii?

Copy and paste relevant sections from manuscript title (include quotes in quotation marks "like this" to indicate direct quotes from your manuscript), or elaborate on this item by providing additional information not in the ms, or briefly explain why the item is not applicable/relevant for your study

"Efficacy of Virtual Agent-Based Digital Intervention (Echo-App V2.0) for Patients with Alcohol Use Disorder: A Randomised Controlled Trial"

1a-iii) Primary condition or target group in the title

Mention primary condition or target group in the title, if any (e.g., “for children with Type I Diabetes”) Example: A Web-based and Mobile Intervention with Telephone Support for Children with Type I Diabetes: Randomized Controlled Trial

	1	2	3	4	5	
subitem not at all important	○	○	○	○	◉	essential

清除所选内容

Does your paper address subitem 1a-iii? *

Copy and paste relevant sections from manuscript title (include quotes in quotation marks "like this" to indicate direct quotes from your manuscript), or elaborate on this item by providing additional information not in the ms, or briefly explain why the item is not applicable/relevant for your study

"Efficacy of Virtual Agent-Based Digital Intervention (Echo-App V2.0) for Patients with Alcohol Use Disorder: A Randomised Controlled Trial"

1b) ABSTRACT: Structured summary of trial design, methods, results, and conclusions

NPT extension: Description of experimental treatment, comparator, care providers, centers, and blinding status.

1b-i) Key features/functionalities/components of the intervention and comparator in the METHODS section of the ABSTRACT

Mention key features/functionalities/components of the intervention and comparator in the abstract. If possible, also mention theories and principles used for designing the site. Keep in mind the needs of systematic reviewers and indexers by including important synonyms. (Note: Only report in the abstract what the main paper is reporting. If this information is missing from the main body of text, consider adding it)

	1	2	3	4	5	
subitem not at all important	○	○	○	○	◉	essential

清除所选内容

Does your paper address subitem 1b-i? *

Copy and paste relevant sections from the manuscript abstract (include quotes in quotation marks "like this" to indicate direct quotes from your manuscript), or elaborate on this item by providing additional information not in the ms, or briefly explain why the item is not applicable/relevant for your study

"Patients in the intervention group received 4-week Echo App V2.0 intervention, consisting of 10 CBT sessions (30-45 minutes each) delivered by VAs. In addition, all patients received TAU (Treatment as usual), including medications, daily 30-minute exercise and psychological education. "

1b-ii) Level of human involvement in the METHODS section of the ABSTRACT

Clarify the level of human involvement in the abstract, e.g., use phrases like “fully automated” vs. “therapist/nurse/care provider/physician-assisted” (mention number and expertise of providers involved, if any). (Note: Only report in the abstract what the main paper is reporting. If this information is missing from the main body of text, consider adding it)

	1	2	3	4	5	
subitem not at all important	○	○	○	◉	○	essential

清除所选内容

Does your paper address subitem 1b-ii?

Copy and paste relevant sections from the manuscript abstract (include quotes in quotation marks "like this" to indicate direct quotes from your manuscript), or elaborate on this item by providing additional information not in the ms, or briefly explain why the item is not applicable/relevant for your study

"In addition, all patients received TAU (Treatment as usual), including medications, daily 30-minute exercise and psychological education. "

1b-iii) Open vs. closed, web-based (self-assessment) vs. face-to-face assessments in the METHODS section of the ABSTRACT

Mention how participants were recruited (online vs. offline), e.g., from an open access website or from a clinic or a closed online user group (closed usergroup trial), and clarify if this was a purely web-based trial, or there were face-to-face components (as part of the intervention or for assessment). Clearly say if outcomes were self-assessed through questionnaires (as common in web-based trials). Note: In traditional offline trials, an open trial (open-label trial) is a type of clinical trial in which both the researchers and participants know which treatment is being administered. To avoid confusion, use “blinded” or “unblinded” to indicated the level of blinding instead of “open”, as “open” in web-based trials usually refers to “open access” (i.e. participants can self-enrol). (Note: Only report in the abstract what the main paper is reporting. If this information is missing from the main body of text, consider adding it)

	1	2	3	4	5	
subitem not at all important	○	○	○	○	◉	essential

清除所选内容

Does your paper address subitem 1b-iii?

Copy and paste relevant sections from the manuscript abstract (include quotes in quotation marks "like this" to indicate direct quotes from your manuscript), or elaborate on this item by providing additional information not in the ms, or briefly explain why the item is not applicable/relevant for your study

“ To compare whether the combination of Echo App V2.0, a VA-based digital psychological intervention and TAU yield greater efficacy than TAU alone, a two-arm, randomized controlled trial with a parallel-group design was conducted in Fuyang No. 3 People's Hospital between April-December 2023. 93 Patients between 18-55 years with diagnosed AUD were randomly assigned to the intervention(n=47) or control group(n=46) using a random number table. ”

1b-iv) RESULTS section in abstract must contain use data

Report number of participants enrolled/assessed in each group, the use/uptake of the intervention (e.g., attrition/adherence metrics, use over time, number of logins etc.), in addition to primary/secondary outcomes. (Note: Only report in the abstract what the main paper is reporting. If this information is missing from the main body of text, consider adding it)

	1	2	3	4	5	
subitem not at all important	○	○	○	○	◉	essential

清除所选内容

Does your paper address subitem 1b-iv?

Copy and paste relevant sections from the manuscript abstract (include quotes in quotation marks "like this" to indicate direct quotes from your manuscript), or elaborate on this item by providing additional information not in the ms, or briefly explain why the item is not applicable/relevant for your study

" All analyses followed the ITT principle. Both groups showed an increase in craving after discharge. The intervention group showed significantly attenuated craving increase at week 8(β = -1.81, 95% CI: [-3.45, -0.16], p = 0.03) and week 16 (β = -1.97, 95% CI: [-3.61, -0.32], p = 0.02). A significant difference in relapse between the two groups at the week 8 follow-up ($\chi2$=4.09, p=0.04). Statistically significant larger improvement in sleep quality was found in the intervention group (β = -3.28, 95% CI: [-5.08, -1.49], p <0.001) at week 4. Perceived stress decreased significantly over time (β = -2.91, 95% CI: [-5.27, -0.56], p = 0.02). Linear regression showed that group and change in PSS significantly predicted craving change at week 8(intervention: β = -2.13, 95% CI: [-4.03, -0.24], p = 0.03 ;PSS: β = 0.15, 95% CI: [0.03, 0.26],p = 0.02) and week 16(intervention:β= -2.24, 95% CI: [-4.07, -0.42], p = 0.02； PSS: p = 0.03).Both group (β = 0.12, 95% CI: [0.01, 0.24], p = 0.03) and abstinence frequency defined as the number of previous quit attempts (β = 0.27, OR = 1.31, 95% CI: [1.06, 1.63], p = 0.01) significantly predict relapse at week 8. No adverse events were reported during the intervention period. "

1b-v) CONCLUSIONS/DISCUSSION in abstract for negative trials

Conclusions/Discussions in abstract for negative trials: Discuss the primary outcome - if the trial is negative (primary outcome not changed), and the intervention was not used, discuss whether negative results are attributable to lack of uptake and discuss reasons. (Note: Only report in the abstract what the main paper is reporting. If this information is missing from the main body of text, consider adding it)

	1	2	3	4	5	
subitem not at all important	◉	○	○	○	○	essential

清除所选内容

Does your paper address subitem 1b-v?

Copy and paste relevant sections from the manuscript abstract (include quotes in quotation marks "like this" to indicate direct quotes from your manuscript), or elaborate on this item by providing additional information not in the ms, or briefly explain why the item is not applicable/relevant for your study

您的回答

INTRODUCTION

2a) In INTRODUCTION: Scientific background and explanation of rationale

2a-i) Problem and the type of system/solution

Describe the problem and the type of system/solution that is object of the study: intended as stand-alone intervention vs. incorporated in broader health care program? Intended for a particular patient population? Goals of the intervention, e.g., being more cost-effective to other interventions, replace or complement other solutions? (Note: Details about the intervention are provided in “Methods” under 5)

	1	2	3	4	5	
subitem not at all important	○	○	○	○	◉	essential

清除所选内容

Does your paper address subitem 2a-i? *

Copy and paste relevant sections from the manuscript (include quotes in quotation marks "like this" to indicate direct quotes from your manuscript), or elaborate on this item by providing additional information not in the ms, or briefly explain why the item is not applicable/relevant for your study

“Echo App V2.0 was designed as a structured, session-based virtual psychotherapist specifically targeting evidence-based mechanisms underlying AUD recovery: psychoeducation on alcohol and craving, CBT-based cognitive restructuring, motivational enhancement, emotion regulation, stress management, sleep quality improvement, and relapse prevention[21, 26-30]. Stress is recognized as a key contributor to alcohol misuse, as individuals often use alcohol to cope with psychological distress[31]. Given this relationship, addressing stress alongside drinking behaviors may enhance intervention efficacy. These therapeutic targets were operationalized across 10 structured sessions (see Table S1).””

2a-ii) Scientific background, rationale: What is known about the (type of) system

Scientific background, rationale: What is known about the (type of) system that is the object of the study (be sure to discuss the use of similar systems for other conditions/diagnoses, if appropiate), motivation for the study, i.e. what are the reasons for and what is the context for this specific study, from which stakeholder viewpoint is the study performed, potential impact of findings [2]. Briefly justify the choice of the comparator.

	1	2	3	4	5	
subitem not at all important	○	○	○	○	◉	essential

清除所选内容

Does your paper address subitem 2a-ii? *

Copy and paste relevant sections from the manuscript (include quotes in quotation marks "like this" to indicate direct quotes from your manuscript), or elaborate on this item by providing additional information not in the ms, or briefly explain why the item is not applicable/relevant for your study

“ However, few studies are based on VA for mental disorders, especially on how to improve the effectiveness of treatment as usual (TAU). The evidence base for digital psychotherapeutic interventions in AUD has advanced considerably in recent years. Web-based CBT program (CBT4CBT) produced greater increases in alcohol abstinence over eight months compared to both clinician-delivered CBT and standard outpatient care, with patients assigned to digital CBT improving their percentage of days abstinent by more than 50%[21]. A 12-week mobile CBT app yielded outcomes comparable to face-to-face CBT for outpatients with alcohol use problems[22]. However, reviews have consistently identified engagement and dropout as major limitations of text-based or minimally interactive platforms, underscoring the need for more immersive delivery formats[17]. VA-based systems with three-dimensional embodiment, voice synthesis, and empathic responsiveness represent a qualitative advance over text-only tools. Therefore, we developed a VA -based digital intervention called the Echo app, which functions as a virtual digital psychotherapist. Preliminary results of the pilot study on individuals with methamphetamine use disorders have been promising. Participants reported a significant improvement in their motivation to abstain from drugs and overall satisfaction with the app[23]. Qualitative feedback from that study also informed revisions to session content and interaction flow for V2.0, including adding physiological monitoring, richer multimedia content (videos, animations), and more emotionally responsive, empathetic interactions[24].Based on the pilot study and evidence-based psychological interventions, we developed Echo App V2.0 for AUD. ””

2b) In INTRODUCTION: Specific objectives or hypotheses

Does your paper address CONSORT subitem 2b? *

Copy and paste relevant sections from the manuscript (include quotes in quotation marks "like this" to indicate direct quotes from your manuscript), or elaborate on this item by providing additional information not in the ms, or briefly explain why the item is not applicable/relevant for your study

“Specifically, this study aimed to assess whether the combination of Echo App V2.0 and TAU yields greater efficacy compared to TAU alone[32]. We hypothesized that the intervention group would demonstrate a significantly greater attenuation of alcohol craving over time and lower relapse rates. Furthermore, we hypothesized that the VA intervention would lead to greater improvements in secondary clinical indicators, including emotional state, sleep quality, and perceived stress. The abstract of the study is shown in Figure 1.”

METHODS

3a) Description of trial design (such as parallel, factorial) including allocation ratio

Does your paper address CONSORT subitem 3a? *

Copy and paste relevant sections from the manuscript (include quotes in quotation marks "like this" to indicate direct quotes from your manuscript), or elaborate on this item by providing additional information not in the ms, or briefly explain why the item is not applicable/relevant for your study

“This study employed a two-arm, single-blind RCT with a parallel-group design and a 1:1 randomization ratio. Participants were randomly allocated to two treatment conditions. The Intervention group received Echo App V2.0 treatment in addition to TAU, while the control group received only TAU. ”

3b) Important changes to methods after trial commencement (such as eligibility criteria), with reasons

Does your paper address CONSORT subitem 3b? *

Copy and paste relevant sections from the manuscript (include quotes in quotation marks "like this" to indicate direct quotes from your manuscript), or elaborate on this item by providing additional information not in the ms, or briefly explain why the item is not applicable/relevant for your study

No change has been made.

Your answer must have a minimum of 25 characters.

3b-i) Bug fixes, Downtimes, Content Changes

Bug fixes, Downtimes, Content Changes: ehealth systems are often dynamic systems. A description of changes to methods therefore also includes important changes made on the intervention or comparator during the trial (e.g., major bug fixes or changes in the functionality or content) (5-iii) and other “unexpected events” that may have influenced study design such as staff changes, system failures/downtimes, etc. [2].

	1	2	3	4	5	
subitem not at all important	◉	○	○	○	○	essential

清除所选内容

Does your paper address subitem 3b-i?

Copy and paste relevant sections from the manuscript (include quotes in quotation marks "like this" to indicate direct quotes from your manuscript), or elaborate on this item by providing additional information not in the ms, or briefly explain why the item is not applicable/relevant for your study

您的回答

4a) Eligibility criteria for participants

Does your paper address CONSORT subitem 4a? *

Copy and paste relevant sections from the manuscript (include quotes in quotation marks "like this" to indicate direct quotes from your manuscript), or elaborate on this item by providing additional information not in the ms, or briefly explain why the item is not applicable/relevant for your study

"Participants were recruited from Fuyang No. 3 People's Hospital between April-December 2023 through advertisement. Inclusion criteria: ages 18-55 years, DSM-5 AUD criteria, alcohol abstinence <3 months[33], computer literacy, and informed consent. Exclusion criteria: comorbid neuropsychiatric diseases, cognitive impairment conditions, family history of mental illness, and inability to complete assessments."

4a-i) Computer / Internet literacy

Computer / Internet literacy is often an implicit "de facto" eligibility criterion - this should be explicitly clarified.

	1	2	3	4	5	
subitem not at all important	○	○	○	○	◉	essential

清除所选内容

Does your paper address subitem 4a-i?

Copy and paste relevant sections from the manuscript (include quotes in quotation marks "like this" to indicate direct quotes from your manuscript), or elaborate on this item by providing additional information not in the ms, or briefly explain why the item is not applicable/relevant for your study

We have mentioned the "computer literacy" as inclusion criteria.

4a-ii) Open vs. closed, web-based vs. face-to-face assessments:

Open vs. closed, web-based vs. face-to-face assessments: Mention how participants were recruited (online vs. offline), e.g., from an open access website or from a clinic, and clarify if this was a purely web-based trial, or there were face-to-face components (as part of the intervention or for assessment), i.e., to what degree got the study team to know the participant. In online-only trials, clarify if participants were quasi-anonymous and whether having multiple identities was possible or whether technical or logistical measures (e.g., cookies, email confirmation, phone calls) were used to detect/prevent these.

	1	2	3	4	5	
subitem not at all important	○	○	○	○	◉	essential

清除所选内容

Does your paper address subitem 4a-ii? *

Copy and paste relevant sections from the manuscript (include quotes in quotation marks "like this" to indicate direct quotes from your manuscript), or elaborate on this item by providing additional information not in the ms, or briefly explain why the item is not applicable/relevant for your study

"Participants were recruited from Fuyang No. 3 People's Hospital between April-December 2023 through advertisement. "

4a-iii) Information giving during recruitment

Information given during recruitment. Specify how participants were briefed for recruitment and in the informed consent procedures (e.g., publish the informed consent documentation as appendix, see also item X26), as this information may have an effect on user self-selection, user expectation and may also bias results.

	1	2	3	4	5	
subitem not at all important	○	○	◉	○	○	essential

清除所选内容

Does your paper address subitem 4a-iii?

Copy and paste relevant sections from the manuscript (include quotes in quotation marks "like this" to indicate direct quotes from your manuscript), or elaborate on this item by providing additional information not in the ms, or briefly explain why the item is not applicable/relevant for your study

"The study protocol was approved by the Institutional Review Board of Shanghai Mental Health Center (REC: 2020-92C2). Written informed consent was obtained from all participants prior to enrollment, clearly explaining the study's purpose, procedures, and their right to withdraw at any time without affecting their standard medical care. To protect privacy and confidentiality, all collected study data were strictly de-identified and stored on secure, encrypted servers accessible only to the core research team. Participants received financial compensation for completing the follow-up assessments. Additionally, no identifiable images or personal details of individual participants are presented in this manuscript or its supplementary materials."

4b) Settings and locations where the data were collected

Does your paper address CONSORT subitem 4b? *

Copy and paste relevant sections from the manuscript (include quotes in quotation marks "like this" to indicate direct quotes from your manuscript), or elaborate on this item by providing additional information not in the ms, or briefly explain why the item is not applicable/relevant for your study

Baseline and T1 are assessment are assessed in hospitalized setting.Follow-up easurement was assessed through a multi-source approach at each follow-up timepoint: structured self-report from participants, corroborating reports from family members where available, and review of available clinical records.

4b-i) Report if outcomes were (self-)assessed through online questionnaires

Clearly report if outcomes were (self-)assessed through online questionnaires (as common in web-based trials) or otherwise.

	1	2	3	4	5	
subitem not at all important	○	○	○	○	◉	essential

清除所选内容

Does your paper address subitem 4b-i? *

Copy and paste relevant sections from the manuscript (include quotes in quotation marks "like this" to indicate direct quotes from your manuscript), or elaborate on this item by providing additional information not in the ms, or briefly explain why the item is not applicable/relevant for your study

Outcomes were (self-)assessed through online questionnaires.

4b-ii) Report how institutional affiliations are displayed

Report how institutional affiliations are displayed to potential participants [on ehealth media], as affiliations with prestigious hospitals or universities may affect volunteer rates, use, and reactions with regards to an intervention.(Not a required item – describe only if this may bias results)

	1	2	3	4	5	
subitem not at all important	○	○	○	○	○	essential

Does your paper address subitem 4b-ii?

Copy and paste relevant sections from the manuscript (include quotes in quotation marks "like this" to indicate direct quotes from your manuscript), or elaborate on this item by providing additional information not in the ms, or briefly explain why the item is not applicable/relevant for your study

您的回答

5) The interventions for each group with sufficient details to allow replication, including how and when they were actually administered

5-i) Mention names, credential, affiliations of the developers, sponsors, and owners

Mention names, credential, affiliations of the developers, sponsors, and owners [6] (if authors/evaluators are owners or developer of the software, this needs to be declared in a “Conflict of interest” section or mentioned elsewhere in the manuscript).

	1	2	3	4	5	
subitem not at all important	○	○	○	○	◉	essential

清除所选内容

Does your paper address subitem 5-i?

Copy and paste relevant sections from the manuscript (include quotes in quotation marks "like this" to indicate direct quotes from your manuscript), or elaborate on this item by providing additional information not in the ms, or briefly explain why the item is not applicable/relevant for your study

"Competing Interests: All authors declare no potential conflicts of interest, including specific financial interests and relationships and affiliations relevant to the subject of this manuscript."

5-ii) Describe the history/development process

Describe the history/development process of the application and previous formative evaluations (e.g., focus groups, usability testing), as these will have an impact on adoption/use rates and help with interpreting results.

	1	2	3	4	5	
subitem not at all important	○	○	○	○	◉	essential

清除所选内容

Does your paper address subitem 5-ii?

Copy and paste relevant sections from the manuscript (include quotes in quotation marks "like this" to indicate direct quotes from your manuscript), or elaborate on this item by providing additional information not in the ms, or briefly explain why the item is not applicable/relevant for your study

"Therefore, we developed a VA -based digital intervention called the Echo app, which functions as a virtual digital psychotherapist. Preliminary results of the pilot study on individuals with methamphetamine use disorders have been promising. Participants reported a significant improvement in their motivation to abstain from drugs and overall satisfaction with the app[23]. Qualitative feedback from that study also informed revisions to session content and interaction flow for V2.0, including adding physiological monitoring, richer multimedia content (videos, animations), and more emotionally responsive, empathetic interactions[24].Based on the pilot study and evidence-based psychological interventions, we developed Echo App V2.0 for AUD.

5-iii) Revisions and updating

Revisions and updating. Clearly mention the date and/or version number of the application/intervention (and comparator, if applicable) evaluated, or describe whether the intervention underwent major changes during the evaluation process, or whether the development and/or content was “frozen” during the trial. Describe dynamic components such as news feeds or changing content which may have an impact on the replicability of the intervention (for unexpected events see item 3b).

	1	2	3	4	5	
subitem not at all important	○	○	○	○	◉	essential

清除所选内容

Does your paper address subitem 5-iii?

Copy and paste relevant sections from the manuscript (include quotes in quotation marks "like this" to indicate direct quotes from your manuscript), or elaborate on this item by providing additional information not in the ms, or briefly explain why the item is not applicable/relevant for your study

"Intervention and comparator
VA-based digital intervention delivered by the Echo App V2.0
Tablet-based intervention consisting of 10 CBT sessions (30-45 minutes each) delivered 2-3 times weekly over 4 weeks during hospitalization. Sessions covered treatment motivation, impulsivity, coping strategies, stress, anxiety, depression, sleep quality, and compulsion through semi-structured human-computer interaction with virtual psychotherapist avatar and audio-guided mindfulness exercises. The virtual character was designed as a 3D-modelled guide to enhance engagement and orientation within the app. Navigation followed a structured pathway: core modules were preplanned to ensure clinical fidelity. Session completion and engagement data were recorded automatically by the app server. Adherence was defined as completing at least 8 of the 10 prescribed sessions. Fidelity was monitored weekly by a supervising clinician who reviewed session logs; technical failures or incomplete sessions were flagged and, where possible, rescheduled within the 4-week inpatient window. The Echo App V2.0 was deployed exclusively on hospital-provided tablets and was accessible only during the inpatient period.
Technical architecture and therapeutic content basis of the Echo App V2.0
The Echo App V2.0 operates on a rule-based, script-driven dialogue architecture. All utterances delivered by the VA are pre-authored text strings rendered at runtime via speech-synthesis technology. The app runs in server mode: the user-side tablet communicates with a central server that stores assessment data and manages session logic across the patient, administrator, and server tiers[32]. The VA does not query an external knowledge base at runtime.
The therapeutic content of the VA was analogous to the training curriculum of a human practitioner and developed by a multidisciplinary team of psychiatrists and clinical psychologists at Shanghai Mental Health Center and was grounded in three evidence-based psychological frameworks recommended for AUD: Motivational Interviewing (MI), Cognitive Behavioral Therapy (CBT), and Mindfulness-Based Interventions (MBI). Specific source materials drawn upon included: (1) the CBT model for substance use disorders as described by Carroll and Kiluk [30], covering cognitive restructuring, high-risk situation identification, and relapse prevention; (2) the MI principles of Miller and Rollnick [34], operationalized to enhance abstinence motivation through decisional-balance exercises; (3) mindfulness techniques for relapse prevention, operationalized in stress management and sitting/mountain meditation; and (4) psychoeducational content on alcohol pharmacology, craving mechanisms, emotion regulation, sleep hygiene, and well-being. Scripts were reviewed and iteratively revised by the clinical team to ensure fidelity to the source CBT and MI frameworks before deployment. The specific themes and content of these 10 sessions with details provided in the Table S1.
Treatment as usual (TAU)
Standardized 4-week hospitalization including medications (benzodiazepines for withdrawal symptoms, tapered over 7-10 days), daily 30-minute exercise (15 minutes Baduanjin, 15 minutes aerobic), and psychological education[35-38]. TAU components were standardized and applied uniformly to all participants in both arms throughout the 4-week inpatient period. No additional co-interventions were permitted or recorded in either group. Medication dosing was individually titrated by treating physicians according to withdrawal severity, but the protocol type remained consistent across participants."

5-iv) Quality assurance methods

Provide information on quality assurance methods to ensure accuracy and quality of information provided [1], if applicable.

	1	2	3	4	5	
subitem not at all important	○	○	○	○	◉	essential

清除所选内容

Does your paper address subitem 5-iv?

Copy and paste relevant sections from the manuscript (include quotes in quotation marks "like this" to indicate direct quotes from your manuscript), or elaborate on this item by providing additional information not in the ms, or briefly explain why the item is not applicable/relevant for your study

"With regard to intervention fidelity, 39 of 47 participants in the intervention group (83.0%) completed all 10 sessions; the mean number of sessions completed was 9.2 (SD = 1.3), with a mean total engagement time of approximately 338 minutes per participant. 6 participants completed fewer than 8 sessions, most commonly due to early discharge (n=4) or device technical issues (n=2). No major protocol deviations were recorded. "

5-v) Ensure replicability by publishing the source code, and/or providing screenshots/screen-capture video, and/or providing flowcharts of the algorithms used

Ensure replicability by publishing the source code, and/or providing screenshots/screen-capture video, and/or providing flowcharts of the algorithms used. Replicability (i.e., other researchers should in principle be able to replicate the study) is a hallmark of scientific reporting.

	1	2	3	4	5	
subitem not at all important	○	○	○	○	◉	essential

清除所选内容

Does your paper address subitem 5-v?

Copy and paste relevant sections from the manuscript (include quotes in quotation marks "like this" to indicate direct quotes from your manuscript), or elaborate on this item by providing additional information not in the ms, or briefly explain why the item is not applicable/relevant for your study

We have screenshots/videos alongside the article.

5-vi) Digital preservation

Digital preservation: Provide the URL of the application, but as the intervention is likely to change or disappear over the course of the years; also make sure the intervention is archived (Internet Archive, webcitation.org, and/or publishing the source code or screenshots/videos alongside the article). As pages behind login screens cannot be archived, consider creating demo pages which are accessible without login.

	1	2	3	4	5	
subitem not at all important	○	○	○	○	◉	essential

清除所选内容

Does your paper address subitem 5-vi?

Copy and paste relevant sections from the manuscript (include quotes in quotation marks "like this" to indicate direct quotes from your manuscript), or elaborate on this item by providing additional information not in the ms, or briefly explain why the item is not applicable/relevant for your study

We have screenshots/videos alongside the article.

5-vii) Access

Access: Describe how participants accessed the application, in what setting/context, if they had to pay (or were paid) or not, whether they had to be a member of specific group. If known, describe how participants obtained “access to the platform and Internet” [1]. To ensure access for editors/reviewers/readers, consider to provide a “backdoor” login account or demo mode for reviewers/readers to explore the application (also important for archiving purposes, see vi).

	1	2	3	4	5	
subitem not at all important	○	○	○	○	◉	essential

清除所选内容

Does your paper address subitem 5-vii? *

Copy and paste relevant sections from the manuscript (include quotes in quotation marks "like this" to indicate direct quotes from your manuscript), or elaborate on this item by providing additional information not in the ms, or briefly explain why the item is not applicable/relevant for your study

"Session completion and engagement data were recorded automatically by the app server. Adherence was defined as completing at least 8 of the 10 prescribed sessions. Fidelity was monitored weekly by a supervising clinician who reviewed session logs; technical failures or incomplete sessions were flagged and, where possible, rescheduled within the 4-week inpatient window. The Echo App V2.0 was deployed exclusively on hospital-provided tablets and was accessible only during the inpatient period."

5-viii) Mode of delivery, features/functionalities/components of the intervention and comparator, and the theoretical framework

Describe mode of delivery, features/functionalities/components of the intervention and comparator, and the theoretical framework [6] used to design them (instructional strategy [1], behaviour change techniques, persuasive features, etc., see e.g., [7, 8] for terminology). This includes an in-depth description of the content (including where it is coming from and who developed it) [1],” whether [and how] it is tailored to individual circumstances and allows users to track their progress and receive feedback” [6]. This also includes a description of communication delivery channels and – if computer-mediated communication is a component – whether communication was synchronous or asynchronous [6]. It also includes information on presentation strategies [1], including page design principles, average amount of text on pages, presence of hyperlinks to other resources, etc. [1].

	1	2	3	4	5	
subitem not at all important	○	○	○	○	◉	essential

清除所选内容

Does your paper address subitem 5-viii? *

Copy and paste relevant sections from the manuscript (include quotes in quotation marks "like this" to indicate direct quotes from your manuscript), or elaborate on this item by providing additional information not in the ms, or briefly explain why the item is not applicable/relevant for your study

"VA-based digital intervention delivered by the Echo App V2.0
Tablet-based intervention consisting of 10 CBT sessions (30-45 minutes each) delivered 2-3 times weekly over 4 weeks during hospitalization. Sessions covered treatment motivation, impulsivity, coping strategies, stress, anxiety, depression, sleep quality, and compulsion through semi-structured human-computer interaction with virtual psychotherapist avatar and audio-guided mindfulness exercises. The virtual character was designed as a 3D-modelled guide to enhance engagement and orientation within the app. Navigation followed a structured pathway: core modules were preplanned to ensure clinical fidelity. Session completion and engagement data were recorded automatically by the app server. Adherence was defined as completing at least 8 of the 10 prescribed sessions. Fidelity was monitored weekly by a supervising clinician who reviewed session logs; technical failures or incomplete sessions were flagged and, where possible, rescheduled within the 4-week inpatient window. The Echo App V2.0 was deployed exclusively on hospital-provided tablets and was accessible only during the inpatient period.
Technical architecture and therapeutic content basis of the Echo App V2.0
The Echo App V2.0 operates on a rule-based, script-driven dialogue architecture. All utterances delivered by the VA are pre-authored text strings rendered at runtime via speech-synthesis technology. The app runs in server mode: the user-side tablet communicates with a central server that stores assessment data and manages session logic across the patient, administrator, and server tiers[32]. The VA does not query an external knowledge base at runtime.
The therapeutic content of the VA was analogous to the training curriculum of a human practitioner and developed by a multidisciplinary team of psychiatrists and clinical psychologists at Shanghai Mental Health Center and was grounded in three evidence-based psychological frameworks recommended for AUD: Motivational Interviewing (MI), Cognitive Behavioral Therapy (CBT), and Mindfulness-Based Interventions (MBI). Specific source materials drawn upon included: (1) the CBT model for substance use disorders as described by Carroll and Kiluk [30], covering cognitive restructuring, high-risk situation identification, and relapse prevention; (2) the MI principles of Miller and Rollnick [34], operationalized to enhance abstinence motivation through decisional-balance exercises; (3) mindfulness techniques for relapse prevention, operationalized in stress management and sitting/mountain meditation; and (4) psychoeducational content on alcohol pharmacology, craving mechanisms, emotion regulation, sleep hygiene, and well-being. Scripts were reviewed and iteratively revised by the clinical team to ensure fidelity to the source CBT and MI frameworks before deployment. The specific themes and content of these 10 sessions with details provided in the Table S1."

5-ix) Describe use parameters

Describe use parameters (e.g., intended “doses” and optimal timing for use). Clarify what instructions or recommendations were given to the user, e.g., regarding timing, frequency, heaviness of use, if any, or was the intervention used ad libitum.

	1	2	3	4	5	
subitem not at all important	○	○	○	○	◉	essential

清除所选内容

Does your paper address subitem 5-ix?

Copy and paste relevant sections from the manuscript (include quotes in quotation marks "like this" to indicate direct quotes from your manuscript), or elaborate on this item by providing additional information not in the ms, or briefly explain why the item is not applicable/relevant for your study

"Tablet-based intervention consisting of 10 CBT sessions (30-45 minutes each) delivered 2-3 times weekly over 4 weeks during hospitalization. Sessions covered treatment motivation, impulsivity, coping strategies, stress, anxiety, depression, sleep quality, and compulsion through semi-structured human-computer interaction with virtual psychotherapist avatar and audio-guided mindfulness exercises. "

5-x) Clarify the level of human involvement

Clarify the level of human involvement (care providers or health professionals, also technical assistance) in the e-intervention or as co-intervention (detail number and expertise of professionals involved, if any, as well as “type of assistance offered, the timing and frequency of the support, how it is initiated, and the medium by which the assistance is delivered”. It may be necessary to distinguish between the level of human involvement required for the trial, and the level of human involvement required for a routine application outside of a RCT setting (discuss under item 21 – generalizability).

	1	2	3	4	5	
subitem not at all important	○	○	○	○	◉	essential

清除所选内容

Does your paper address subitem 5-x?

Copy and paste relevant sections from the manuscript (include quotes in quotation marks "like this" to indicate direct quotes from your manuscript), or elaborate on this item by providing additional information not in the ms, or briefly explain why the item is not applicable/relevant for your study

"Treatment as usual (TAU)
Standardized 4-week hospitalization including medications (benzodiazepines for withdrawal symptoms, tapered over 7-10 days), daily 30-minute exercise (15 minutes Baduanjin, 15 minutes aerobic), and psychological education[35-38]. TAU components were standardized and applied uniformly to all participants in both arms throughout the 4-week inpatient period. No additional co-interventions were permitted or recorded in either group. Medication dosing was individually titrated by treating physicians according to withdrawal severity, but the protocol type remained consistent across participants."

5-xi) Report any prompts/reminders used

Report any prompts/reminders used: Clarify if there were prompts (letters, emails, phone calls, SMS) to use the application, what triggered them, frequency etc. It may be necessary to distinguish between the level of prompts/reminders required for the trial, and the level of prompts/reminders for a routine application outside of a RCT setting (discuss under item 21 – generalizability).

	1	2	3	4	5	
subitem not at all important	○	○	○	○	◉	essential

清除所选内容

Does your paper address subitem 5-xi? *

Copy and paste relevant sections from the manuscript (include quotes in quotation marks "like this" to indicate direct quotes from your manuscript), or elaborate on this item by providing additional information not in the ms, or briefly explain why the item is not applicable/relevant for your study

"Adverse events and safety
Adverse events and participant safety were monitored throughout the study. No serious adverse events related to the intervention were recorded. Participants showing signs of clinical deterioration were referred to the clinical team for evaluation. No participants were withdrawn from the study due to intervention-related harm. Digital interactions were reviewed by the clinical supervisor weekly to identify any unexpected distress."

5-xii) Describe any co-interventions (incl. training/support)

Describe any co-interventions (incl. training/support): Clearly state any interventions that are provided in addition to the targeted eHealth intervention, as ehealth intervention may not be designed as stand-alone intervention. This includes training sessions and support [1]. It may be necessary to distinguish between the level of training required for the trial, and the level of training for a routine application outside of a RCT setting (discuss under item 21 – generalizability.

	1	2	3	4	5	
subitem not at all important	○	○	○	○	○	essential

Does your paper address subitem 5-xii? *

Copy and paste relevant sections from the manuscript (include quotes in quotation marks "like this" to indicate direct quotes from your manuscript), or elaborate on this item by providing additional information not in the ms, or briefly explain why the item is not applicable/relevant for your study

"Treatment as usual (TAU)
Standardized 4-week hospitalization including medications (benzodiazepines for withdrawal symptoms, tapered over 7-10 days), daily 30-minute exercise (15 minutes Baduanjin, 15 minutes aerobic), and psychological education[35-38]. TAU components were standardized and applied uniformly to all participants in both arms throughout the 4-week inpatient period. No additional co-interventions were permitted or recorded in either group. Medication dosing was individually titrated by treating physicians according to withdrawal severity, but the protocol type remained consistent across participants."

6a) Completely defined pre-specified primary and secondary outcome measures, including how and when they were assessed

Does your paper address CONSORT subitem 6a? *

Copy and paste relevant sections from the manuscript (include quotes in quotation marks "like this" to indicate direct quotes from your manuscript), or elaborate on this item by providing additional information not in the ms, or briefly explain why the item is not applicable/relevant for your study

"The primary outcome was alcohol craving measured by the Visual Analogue Scale (VAS) across four time points (baseline, week 4, week 8, and week 16). A 10-centimeter horizontal line was presented to participants, from left endpoint labeled as “no craving at all” (0) to right endpoint as “extreme craving” (10). A change of 1.0 point or greater on the VAS has been proposed as a clinically meaningful difference in craving intensity in substance use populations[39].The primary hypothesis concerned whether the trajectory of craving over time differed between the intervention and control groups.
Secondary outcomes
Secondary outcome is alcohol relapse at follow-up assessment after participants were discharged from the hospital. Alcohol relapse was defined whether the participants drank 4 or more (female), or 5 or more (male) standard drinks in the last 14 days[40]. We also define patients lost to follow-up as relapse because previous studies have shown that when patients relapse, they usually avoid contact with therapists or researchers due to a sense of shame or frustration[41]. This conservative, intent-to-treat-consistent approach is commonly used in substance use trials to avoid overestimating abstinence rates.
Other measurements include treatment motivation evaluated with the Stages of Change Readiness and Treatment Eagerness Scale (SOCRATES), anxiety measured via the Generalized Anxiety Disorder-7 (GAD-7), depression evaluated by the Patient Health Questionnaire-9 (PHQ-9), stress gauged through the Perceived Stress Scale (PSS) and sleep quality assessed by the Pittsburgh Sleep Quality Index (PSQI). The Chinese versions of the scales have good reliability and validity. The Chinese versions of all secondary outcome scales have been validated in Chinese populations and demonstrate good psychometric properties: GAD-7 (Cronbach's α = 0.90)[42],PHQ-9 (α = 0.86)[43], PSQI (α = 0.73)[44],PSS (α = 0.85)[45],and SOCRATES[46]. No pre-specified clinical thresholds were applied to secondary outcomes and all were analyzed as continuous variables."

6a-i) Online questionnaires: describe if they were validated for online use and apply CHERRIES items to describe how the questionnaires were designed/deployed

If outcomes were obtained through online questionnaires, describe if they were validated for online use and apply CHERRIES items to describe how the questionnaires were designed/deployed [9].

	1	2	3	4	5	
subitem not at all important	◉	○	○	○	○	essential

清除所选内容

Does your paper address subitem 6a-i?

Copy and paste relevant sections from manuscript text

The validity of the questionnaires has been proven:" The Chinese versions of all secondary outcome scales have been validated in Chinese populations and demonstrate good psychometric properties: GAD-7 (Cronbach's α = 0.90)[42],PHQ-9 (α = 0.86)[43], PSQI (α = 0.73)[44],PSS (α = 0.85)[45],and SOCRATES[46]. "

6a-ii) Describe whether and how "use" (including intensity of use/dosage) was defined/measured/monitored

Describe whether and how "use" (including intensity of use/dosage) was defined/measured/monitored (logins, logfile analysis, etc.). Use/adoption metrics are important process outcomes that should be reported in any ehealth trial.

	1	2	3	4	5	
subitem not at all important	○	○	○	○	◉	essential

清除所选内容

Does your paper address subitem 6a-ii?

Copy and paste relevant sections from manuscript text

"The primary outcome was alcohol craving measured by the Visual Analogue Scale (VAS) across four time points (baseline, week 4, week 8, and week 16)." and "Secondary outcome is alcohol relapse at follow-up assessment after participants were discharged from the hospital." and "Other measurements include treatment motivation evaluated with the Stages of Change Readiness and Treatment Eagerness Scale (SOCRATES), anxiety measured via the Generalized Anxiety Disorder-7 (GAD-7), depression evaluated by the Patient Health Questionnaire-9 (PHQ-9), stress gauged through the Perceived Stress Scale (PSS) and sleep quality assessed by the Pittsburgh Sleep Quality Index (PSQI)."

6a-iii) Describe whether, how, and when qualitative feedback from participants was obtained

Describe whether, how, and when qualitative feedback from participants was obtained (e.g., through emails, feedback forms, interviews, focus groups).

	1	2	3	4	5	
subitem not at all important	◉	○	○	○	○	essential

清除所选内容

Does your paper address subitem 6a-iii?

Copy and paste relevant sections from manuscript text

您的回答

6b) Any changes to trial outcomes after the trial commenced, with reasons

Does your paper address CONSORT subitem 6b? *

Copy and paste relevant sections from the manuscript (include quotes in quotation marks "like this" to indicate direct quotes from your manuscript), or elaborate on this item by providing additional information not in the ms, or briefly explain why the item is not applicable/relevant for your study

No qualitative feedback was ontained form this RCT.

7a) How sample size was determined

NPT: When applicable, details of whether and how the clustering by care provides or centers was addressed

7a-i) Describe whether and how expected attrition was taken into account when calculating the sample size

Describe whether and how expected attrition was taken into account when calculating the sample size.

	1	2	3	4	5	
subitem not at all important	○	○	○	○	◉	essential

清除所选内容

Does your paper address subitem 7a-i?

Copy and paste relevant sections from manuscript title (include quotes in quotation marks "like this" to indicate direct quotes from your manuscript), or elaborate on this item by providing additional information not in the ms, or briefly explain why the item is not applicable/relevant for your study

"Drawing from prior literature of innovative interventions on individuals with AUD[9], to achieve 80% statistical power to detect a medium effect size (Cohen's d = 0.56) in the trajectory of craving scores between intervention and control groups, assuming 4 measurement time points with correlation of 0.6 between repeated measures, a total of 35 participants were required for each group. In anticipation of 30% dropout rate, a total sample size of 100 participants were required, 50 participants per group."

7b) When applicable, explanation of any interim analyses and stopping guidelines

Does your paper address CONSORT subitem 7b? *

Copy and paste relevant sections from the manuscript (include quotes in quotation marks "like this" to indicate direct quotes from your manuscript), or elaborate on this item by providing additional information not in the ms, or briefly explain why the item is not applicable/relevant for your study

"Adverse events and safety
Adverse events and participant safety were monitored throughout the study. No serious adverse events related to the intervention were recorded. Participants showing signs of clinical deterioration were referred to the clinical team for evaluation. No participants were withdrawn from the study due to intervention-related harm. Digital interactions were reviewed by the clinical supervisor weekly to identify any unexpected distress."

8a) Method used to generate the random allocation sequence
NPT: When applicable, how care providers were allocated to each trial group

Does your paper address CONSORT subitem 8a? *

Copy and paste relevant sections from the manuscript (include quotes in quotation marks "like this" to indicate direct quotes from your manuscript), or elaborate on this item by providing additional information not in the ms, or briefly explain why the item is not applicable/relevant for your study

"A total of 93 participants were randomly assigned (1:1) to intervention (Echo App V2.0 + TAU, n=47) or control (TAU, n=46) groups using a random number table. The random allocation sequence was generated by an independent statistician using a computer-generated random number table prior to trial commencement. Participants were enrolled by treating clinicians. Group assignment was performed by a designated research coordinator who was not involved in participant recruitment or outcome assessment. Allocation concealment was maintained using sequentially numbered, opaque sealed envelopes, which were opened only after a participant had provided informed consent and been formally enrolled. Given the nature of the intervention, neither participants nor treating clinicians could be blinded to group assignment. To mitigate expectation effects, both groups received equivalent clinician contact time. Control participants received standard psychological education as part of TAU, ensuring that the between-group comparison reflected the specific contribution of the Echo App V2.0 beyond general attention and psychoeducation. Researchers responsible for outcome assessment and data analysis were blinded to group allocation throughout the study period. Researchers responsible for assessing outcomes and analyzing data were blinded to participants' group allocations. "

8b) Type of randomisation; details of any restriction (such as blocking and block size)

Does your paper address CONSORT subitem 8b? *

Copy and paste relevant sections from the manuscript (include quotes in quotation marks "like this" to indicate direct quotes from your manuscript), or elaborate on this item by providing additional information not in the ms, or briefly explain why the item is not applicable/relevant for your study

"A total of 93 participants were randomly assigned (1:1) to intervention (Echo App V2.0 + TAU, n=47) or control (TAU, n=46) groups using a random number table. The random allocation sequence was generated by an independent statistician using a computer-generated random number table prior to trial commencement. Participants were enrolled by treating clinicians. Group assignment was performed by a designated research coordinator who was not involved in participant recruitment or outcome assessment. Allocation concealment was maintained using sequentially numbered, opaque sealed envelopes, which were opened only after a participant had provided informed consent and been formally enrolled. Given the nature of the intervention, neither participants nor treating clinicians could be blinded to group assignment. To mitigate expectation effects, both groups received equivalent clinician contact time. Control participants received standard psychological education as part of TAU, ensuring that the between-group comparison reflected the specific contribution of the Echo App V2.0 beyond general attention and psychoeducation. Researchers responsible for outcome assessment and data analysis were blinded to group allocation throughout the study period. Researchers responsible for assessing outcomes and analyzing data were blinded to participants' group allocations. "

9) Mechanism used to implement the random allocation sequence (such as sequentially numbered containers), describing any steps taken to conceal the sequence until interventions were assigned

Does your paper address CONSORT subitem 9? *

Copy and paste relevant sections from the manuscript (include quotes in quotation marks "like this" to indicate direct quotes from your manuscript), or elaborate on this item by providing additional information not in the ms, or briefly explain why the item is not applicable/relevant for your study

"A total of 93 participants were randomly assigned (1:1) to intervention (Echo App V2.0 + TAU, n=47) or control (TAU, n=46) groups using a random number table. The random allocation sequence was generated by an independent statistician using a computer-generated random number table prior to trial commencement. Participants were enrolled by treating clinicians. Group assignment was performed by a designated research coordinator who was not involved in participant recruitment or outcome assessment. Allocation concealment was maintained using sequentially numbered, opaque sealed envelopes, which were opened only after a participant had provided informed consent and been formally enrolled. Given the nature of the intervention, neither participants nor treating clinicians could be blinded to group assignment. To mitigate expectation effects, both groups received equivalent clinician contact time. Control participants received standard psychological education as part of TAU, ensuring that the between-group comparison reflected the specific contribution of the Echo App V2.0 beyond general attention and psychoeducation. Researchers responsible for outcome assessment and data analysis were blinded to group allocation throughout the study period. Researchers responsible for assessing outcomes and analyzing data were blinded to participants' group allocations. "

10) Who generated the random allocation sequence, who enrolled participants, and who assigned participants to interventions

Does your paper address CONSORT subitem 10? *

Copy and paste relevant sections from the manuscript (include quotes in quotation marks "like this" to indicate direct quotes from your manuscript), or elaborate on this item by providing additional information not in the ms, or briefly explain why the item is not applicable/relevant for your study

"A total of 93 participants were randomly assigned (1:1) to intervention (Echo App V2.0 + TAU, n=47) or control (TAU, n=46) groups using a random number table. The random allocation sequence was generated by an independent statistician using a computer-generated random number table prior to trial commencement. Participants were enrolled by treating clinicians. Group assignment was performed by a designated research coordinator who was not involved in participant recruitment or outcome assessment. Allocation concealment was maintained using sequentially numbered, opaque sealed envelopes, which were opened only after a participant had provided informed consent and been formally enrolled. Given the nature of the intervention, neither participants nor treating clinicians could be blinded to group assignment. To mitigate expectation effects, both groups received equivalent clinician contact time. Control participants received standard psychological education as part of TAU, ensuring that the between-group comparison reflected the specific contribution of the Echo App V2.0 beyond general attention and psychoeducation. Researchers responsible for outcome assessment and data analysis were blinded to group allocation throughout the study period. Researchers responsible for assessing outcomes and analyzing data were blinded to participants' group allocations. "

11a) If done, who was blinded after assignment to interventions (for example, participants, care providers, those assessing outcomes) and how

NPT: Whether or not administering co-interventions were blinded to group assignment

11a-i) Specify who was blinded, and who wasn't

Specify who was blinded, and who wasn't. Usually, in web-based trials it is not possible to blind the participants [1, 3] (this should be clearly acknowledged), but it may be possible to blind outcome assessors, those doing data analysis or those administering co-interventions (if any).

	1	2	3	4	5	
subitem not at all important	○	○	○	○	◉	essential

清除所选内容

Does your paper address subitem 11a-i? *

Copy and paste relevant sections from the manuscript (include quotes in quotation marks "like this" to indicate direct quotes from your manuscript), or elaborate on this item by providing additional information not in the ms, or briefly explain why the item is not applicable/relevant for your study

"A total of 93 participants were randomly assigned (1:1) to intervention (Echo App V2.0 + TAU, n=47) or control (TAU, n=46) groups using a random number table. The random allocation sequence was generated by an independent statistician using a computer-generated random number table prior to trial commencement. Participants were enrolled by treating clinicians. Group assignment was performed by a designated research coordinator who was not involved in participant recruitment or outcome assessment. Allocation concealment was maintained using sequentially numbered, opaque sealed envelopes, which were opened only after a participant had provided informed consent and been formally enrolled. Given the nature of the intervention, neither participants nor treating clinicians could be blinded to group assignment. To mitigate expectation effects, both groups received equivalent clinician contact time. Control participants received standard psychological education as part of TAU, ensuring that the between-group comparison reflected the specific contribution of the Echo App V2.0 beyond general attention and psychoeducation. Researchers responsible for outcome assessment and data analysis were blinded to group allocation throughout the study period. Researchers responsible for assessing outcomes and analyzing data were blinded to participants' group allocations. "

11a-ii) Discuss e.g., whether participants knew which intervention was the "intervention of interest" and which one was the "comparator"

Informed consent procedures (4a-ii) can create biases and certain expectations - discuss e.g., whether participants knew which intervention was the "intervention of interest" and which one was the "comparator".

	1	2	3	4	5	
subitem not at all important	○	○	○	○	◉	essential

清除所选内容

Does your paper address subitem 11a-ii?

Copy and paste relevant sections from the manuscript (include quotes in quotation marks "like this" to indicate direct quotes from your manuscript), or elaborate on this item by providing additional information not in the ms, or briefly explain why the item is not applicable/relevant for your study

Participants knew which intervention was the "intervention of interest" because they received intervention from an APP. ".A total of 93 participants were randomly assigned (1:1) to intervention (Echo App V2.0 + TAU, n=47) or control (TAU, n=46) groups using a random number table. The random allocation sequence was generated by an independent statistician using a computer-generated random number table prior to trial commencement. Participants were enrolled by treating clinicians. Group assignment was performed by a designated research coordinator who was not involved in participant recruitment or outcome assessment. Allocation concealment was maintained using sequentially numbered, opaque sealed envelopes, which were opened only after a participant had provided informed consent and been formally enrolled. Given the nature of the intervention, neither participants nor treating clinicians could be blinded to group assignment. To mitigate expectation effects, both groups received equivalent clinician contact time. Control participants received standard psychological education as part of TAU, ensuring that the between-group comparison reflected the specific contribution of the Echo App V2.0 beyond general attention and psychoeducation. Researchers responsible for outcome assessment and data analysis were blinded to group allocation throughout the study period. Researchers responsible for assessing outcomes and analyzing data were blinded to participants' group allocations. "

11b) If relevant, description of the similarity of interventions

(this item is usually not relevant for ehealth trials as it refers to similarity of a placebo or sham intervention to a active medication/intervention)

Does your paper address CONSORT subitem 11b? *

Copy and paste relevant sections from the manuscript (include quotes in quotation marks "like this" to indicate direct quotes from your manuscript), or elaborate on this item by providing additional information not in the ms, or briefly explain why the item is not applicable/relevant for your study

"TAU components were standardized and applied uniformly to all participants in both arms throughout the 4-week inpatient period. No additional co-interventions were permitted or recorded in either group. Medication dosing was individually titrated by treating physicians according to withdrawal severity, but the protocol type remained consistent across participants."

12a) Statistical methods used to compare groups for primary and secondary outcomes

NPT: When applicable, details of whether and how the clustering by care providers or centers was addressed

Does your paper address CONSORT subitem 12a? *

Copy and paste relevant sections from the manuscript (include quotes in quotation marks "like this" to indicate direct quotes from your manuscript), or elaborate on this item by providing additional information not in the ms, or briefly explain why the item is not applicable/relevant for your study

"R software was used with a two-sided α=0.05 and 95% CI（confidence interval）. Baseline variables were summarized by mean (SD) or frequency (%). Independent t-tests were applied to normally distributed data. All analyses followed the ITT principle. Generalized linear mixed models(GLMMs) assessed changes in VAS and secondary outcomes (PSQI, GAD-7, PHQ-9, PSS, SCORATES), with fixed effects for group, time, and group×time. For craving, we used the Last Observation Carried Forward (LOCF) for imputation. For the relapse outcome, missing data were handled in which all participants with missing outcome data were classified as having relapsed. Chi-square analysis was conducted to evaluate the response of relapse/remission.

For sensitivity analyses, two approaches were employed to assess the robustness of findings. For alcohol craving, missing data were addressed using multiple imputation (MI), with 50 imputation datasets generated using predictive mean matching and pooled according to Rubin's rules[47]. A tipping-point analysis was further conducted to nullify the observed treatment effect, thereby evaluating the robustness of the craving findings[48]. For relapse data, we firstly calculated respondents only situation(missing=missing: participants lost to follow up were not included in the analysis).Then best-case and worst-case scenario analyses were performed: in the best-case scenario, all missing values in the treatment group were imputed as non-relapse and all missing values in the control group as relapse; the worst-case scenario applied the reverse assumption[49].

We conducted linear regression model to explore the influence factors on craving. Variables such as intervention assignment and alcohol use history were also selected. Additionally, all other potential variables were analyzed individually to preliminarily identify those significantly associated with craving for alcohol at follow-up. Variables with a p-value less than 0.2 were selected as candidate variables for inclusion in the multivariable model. Finally, those selected variables were put into stepwise regression determine the final model. We conducted logistic model to explore the influence factors on relapse/remission using the same variable selection procedure."

12a-i) Imputation techniques to deal with attrition / missing values

Imputation techniques to deal with attrition / missing values: Not all participants will use the intervention/comparator as intended and attrition is typically high in ehealth trials. Specify how participants who did not use the application or dropped out from the trial were treated in the statistical analysis (a complete case analysis is strongly discouraged, and simple imputation techniques such as LOCF may also be problematic [4]).

	1	2	3	4	5	
subitem not at all important	○	○	○	○	◉	essential

清除所选内容

Does your paper address subitem 12a-i? *

Copy and paste relevant sections from the manuscript (include quotes in quotation marks "like this" to indicate direct quotes from your manuscript), or elaborate on this item by providing additional information not in the ms, or briefly explain why the item is not applicable/relevant for your study

"For craving, we used the Last Observation Carried Forward (LOCF) for imputation. For the relapse outcome, missing data were handled in which all participants with missing outcome data were classified as having relapsed.".We also conducted sentivity analysis:"For sensitivity analyses, two approaches were employed to assess the robustness of findings. For alcohol craving, missing data were addressed using multiple imputation (MI), with 50 imputation datasets generated using predictive mean matching and pooled according to Rubin's rules[47]. A tipping-point analysis was further conducted to nullify the observed treatment effect, thereby evaluating the robustness of the craving findings[48]. For relapse data, we firstly calculated respondents only situation(missing=missing: participants lost to follow up were not included in the analysis).Then best-case and worst-case scenario analyses were performed: in the best-case scenario, all missing values in the treatment group were imputed as non-relapse and all missing values in the control group as relapse; the worst-case scenario applied the reverse assumption[49]. "

12b) Methods for additional analyses, such as subgroup analyses and adjusted analyses

Does your paper address CONSORT subitem 12b? *

Copy and paste relevant sections from the manuscript (include quotes in quotation marks "like this" to indicate direct quotes from your manuscript), or elaborate on this item by providing additional information not in the ms, or briefly explain why the item is not applicable/relevant for your study

"We conducted linear regression model to explore the influence factors on craving. Variables such as intervention assignment and alcohol use history were also selected. Additionally, all other potential variables were analyzed individually to preliminarily identify those significantly associated with craving for alcohol at follow-up. Variables with a p-value less than 0.2 were selected as candidate variables for inclusion in the multivariable model. Finally, those selected variables were put into stepwise regression determine the final model. We conducted logistic model to explore the influence factors on relapse/remission using the same variable selection procedure."

X26) REB/IRB Approval and Ethical Considerations [recommended as subheading under "Methods"] (not a CONSORT item)

X26-i) Comment on ethics committee approval

	1	2	3	4	5	
subitem not at all important	○	○	○	○	●	essential

清除所选内容

Does your paper address subitem X26-i?

Copy and paste relevant sections from the manuscript (include quotes in quotation marks "like this" to indicate direct quotes from your manuscript), or elaborate on this item by providing additional information not in the ms, or briefly explain why the item is not applicable/relevant for your study

"Ethical considerations
The study protocol was approved by the Institutional Review Board of Shanghai Mental Health Center (REC: 2020-92C2). Written informed consent was obtained from all participants prior to enrollment, clearly explaining the study's purpose, procedures, and their right to withdraw at any time without affecting their standard medical care. To protect privacy and confidentiality, all collected study data were strictly de-identified and stored on secure, encrypted servers accessible only to the core research team. Participants received financial compensation for completing the follow-up assessments. Additionally, no identifiable images or personal details of individual participants are presented in this manuscript or its supplementary materials."

x26-ii) Outline informed consent procedures

Outline informed consent procedures e.g., if consent was obtained offline or online (how? Checkbox, etc.?), and what information was provided (see 4a-ii). See [6] for some items to be included in informed consent documents.

	1	2	3	4	5	
subitem not at all important	○	○	○	○	◉	essential

清除所选内容

Does your paper address subitem X26-ii?

Copy and paste relevant sections from the manuscript (include quotes in quotation marks "like this" to indicate direct quotes from your manuscript), or elaborate on this item by providing additional information not in the ms, or briefly explain why the item is not applicable/relevant for your study

"Ethical considerations
The study protocol was approved by the Institutional Review Board of Shanghai Mental Health Center (REC: 2020-92C2). Written informed consent was obtained from all participants prior to enrollment, clearly explaining the study's purpose, procedures, and their right to withdraw at any time without affecting their standard medical care. To protect privacy and confidentiality, all collected study data were strictly de-identified and stored on secure, encrypted servers accessible only to the core research team. Participants received financial compensation for completing the follow-up assessments. Additionally, no identifiable images or personal details of individual participants are presented in this manuscript or its supplementary materials."

X26-iii) Safety and security procedures

Safety and security procedures, incl. privacy considerations, and any steps taken to reduce the likelihood or detection of harm (e.g., education and training, availability of a hotline)

	1	2	3	4	5	
subitem not at all important	○	○	○	○	●	essential

清除所选内容

Does your paper address subitem X26-iii?

Copy and paste relevant sections from the manuscript (include quotes in quotation marks "like this" to indicate direct quotes from your manuscript), or elaborate on this item by providing additional information not in the ms, or briefly explain why the item is not applicable/relevant for your study

"Adverse events and safety
Adverse events and participant safety were monitored throughout the study. No serious adverse events related to the intervention were recorded. Participants showing signs of clinical deterioration were referred to the clinical team for evaluation. No participants were withdrawn from the study due to intervention-related harm. Digital interactions were reviewed by the clinical supervisor weekly to identify any unexpected distress."

RESULTS

13a) For each group, the numbers of participants who were randomly assigned, received intended treatment, and were analysed for the primary outcome
NPT: The number of care providers or centers performing the intervention in each group and the number of patients treated by each care provider in each center

Does your paper address CONSORT subitem 13a? *

Copy and paste relevant sections from the manuscript (include quotes in quotation marks "like this" to indicate direct quotes from your manuscript), or elaborate on this item by providing additional information not in the ms, or briefly explain why the item is not applicable/relevant for your study

"The specific research process is shown in Figure 2. 15 of 115 participants assessed for eligibility were excluded. Of these, 7 subsequently withdrew consent, and the remaining 93 participants were randomized to the intervention group (TAU + Echo App V2.0, n = 47) or the control group (TAU only, n = 46). 13 participants and 29 participants dropped out at T2 (dropout rate:14%) and T3(dropout rate:31%) respectively. With regard to intervention fidelity, 39 of 47 participants in the intervention group (83.0%) completed all 10 sessions; the mean number of sessions completed was 9.2 (SD = 1.3), with a mean total engagement time of approximately 338 minutes per participant. 6 participants completed fewer than 8 sessions, most commonly due to early discharge (n=4) or device technical issues (n=2). No major protocol deviations were recorded. The total 93 participants were included in ITT analyses."

13b) For each group, losses and exclusions after randomisation, together with reasons

Does your paper address CONSORT subitem 13b? (NOTE: Preferably, this is shown in a CONSORT flow diagram) *

Copy and paste relevant sections from the manuscript (include quotes in quotation marks "like this" to indicate direct quotes from your manuscript), or elaborate on this item by providing additional information not in the ms, or briefly explain why the item is not applicable/relevant for your study

"The specific research process is shown in Figure 2. 15 of 115 participants assessed for eligibility were excluded. Of these, 7 subsequently withdrew consent, and the remaining 93 participants were randomized to the intervention group (TAU + Echo App V2.0, n = 47) or the control group (TAU only, n = 46). 13 participants and 29 participants dropped out at T2 (dropout rate:14%) and T3(dropout rate:31%) respectively. With regard to intervention fidelity, 39 of 47 participants in the intervention group (83.0%) completed all 10 sessions; the mean number of sessions completed was 9.2 (SD = 1.3), with a mean total engagement time of approximately 338 minutes per participant. 6 participants completed fewer than 8 sessions, most commonly due to early discharge (n=4) or device technical issues (n=2). No major protocol deviations were recorded. The total 93 participants were included in ITT analyses.'

13b-i) Attrition diagram

Strongly recommended: An attrition diagram (e.g., proportion of participants still logging in or using the intervention/comparator in each group plotted over time, similar to a survival curve) or other figures or tables demonstrating usage/dose/engagement.

	1	2	3	4	5	
subitem not at all important	○	○	○	○	◉	essential

清除所选内容

Does your paper address subitem 13b-i?

Copy and paste relevant sections from the manuscript or cite the figure number if applicable (include quotes in quotation marks "like this" to indicate direct quotes from your manuscript), or elaborate on this item by providing additional information not in the ms, or briefly explain why the item is not applicable/relevant for your study

"Figure 2. CONSORT flow diagram of participant enrollment, allocation, follow-up, and analysis. Of 115 adults screened, 93 eligible participants aged 18–55 with DSM-5 AUD were randomized (1:1) to the intervention group (n=47) or control group (n=46). Follow-up assessments were conducted at week 4 (T1; inpatient), week 8 (T2; post-discharge), and week 16 (T3; post-discharge). Dropout rates were 14% at T2 and 31% at T3. All 93 participants were included in intention-to-treat (ITT) analyses."

14a) Dates defining the periods of recruitment and follow-up

Does your paper address CONSORT subitem 14a? *

Copy and paste relevant sections from the manuscript (include quotes in quotation marks "like this" to indicate direct quotes from your manuscript), or elaborate on this item by providing additional information not in the ms, or briefly explain why the item is not applicable/relevant for your study

"Participants were recruited from Fuyang No. 3 People's Hospital between April-December 2023 through advertisement. "
"The primary outcome was alcohol craving measured by the Visual Analogue Scale (VAS) across four time points (baseline, week 4, week 8, and week 16)."
"Secondary outcome is alcohol relapse at follow-up assessment after participants were discharged from the hospital. "

14a-i) Indicate if critical “secular events” fell into the study period

Indicate if critical “secular events” fell into the study period, e.g., significant changes in Internet resources available or “changes in computer hardware or Internet delivery resources”

	1	2	3	4	5	
subitem not at all important	(●)	( )	( )	( )	( )	essential

清除所选内容

Does your paper address subitem 14a-i?

Copy and paste relevant sections from the manuscript (include quotes in quotation marks "like this" to indicate direct quotes from your manuscript), or elaborate on this item by providing additional information not in the ms, or briefly explain why the item is not applicable/relevant for your study

您的回答

14b) Why the trial ended or was stopped (early)

Does your paper address CONSORT subitem 14b? *

Copy and paste relevant sections from the manuscript (include quotes in quotation marks "like this" to indicate direct quotes from your manuscript), or elaborate on this item by providing additional information not in the ms, or briefly explain why the item is not applicable/relevant for your study

The trial was not ended or stopped (early).

15) A table showing baseline demographic and clinical characteristics for each group

NPT: When applicable, a description of care providers (case volume, qualification, expertise, etc.) and centers (volume) in each group

Does your paper address CONSORT subitem 15? *

Copy and paste relevant sections from the manuscript (include quotes in quotation marks "like this" to indicate direct quotes from your manuscript), or elaborate on this item by providing additional information not in the ms, or briefly explain why the item is not applicable/relevant for your study

This table is shown in"Table 1. Baseline sociodemographic and clinical characteristics "

15-i) Report demographics associated with digital divide issues

In ehealth trials it is particularly important to report demographics associated with digital divide issues, such as age, education, gender, social-economic status, computer/Internet/ehealth literacy of the participants, if known.

	1	2	3	4	5	
subitem not at all important	○	○	○	○	◉	essential

清除所选内容

Does your paper address subitem 15-i? *

Copy and paste relevant sections from the manuscript (include quotes in quotation marks "like this" to indicate direct quotes from your manuscript), or elaborate on this item by providing additional information not in the ms, or briefly explain why the item is not applicable/relevant for your study

This table is shown in"Table 1. Baseline sociodemographic and clinical characteristics "

16) For each group, number of participants (denominator) included in each analysis and whether the analysis was by original assigned groups

16-i) Report multiple “denominators” and provide definitions

Report multiple “denominators” and provide definitions: Report N’s (and effect sizes) “across a range of study participation [and use] thresholds” [1], e.g., N exposed, N consented, N used more than x times, N used more than y weeks, N participants “used” the intervention/comparator at specific pre-defined time points of interest (in absolute and relative numbers per group). Always clearly define “use” of the intervention.

	1	2	3	4	5	
subitem not at all important	○	○	○	○	◉	essential

清除所选内容

Does your paper address subitem 16-i? *

Copy and paste relevant sections from the manuscript (include quotes in quotation marks "like this" to indicate direct quotes from your manuscript), or elaborate on this item by providing additional information not in the ms, or briefly explain why the item is not applicable/relevant for your study

"The specific research process is shown in Figure 2. 15 of 115 participants assessed for eligibility were excluded. Of these, 7 subsequently withdrew consent, and the remaining 93 participants were randomized to the intervention group (TAU + Echo App V2.0, n = 47) or the control group (TAU only, n = 46). 13 participants and 29 participants dropped out at T2 (dropout rate:14%) and T3(dropout rate:31%) respectively. With regard to intervention fidelity, 39 of 47 participants in the intervention group (83.0%) completed all 10 sessions; the mean number of sessions completed was 9.2 (SD = 1.3), with a mean total engagement time of approximately 338 minutes per participant. 6 participants completed fewer than 8 sessions, most commonly due to early discharge (n=4) or device technical issues (n=2). No major protocol deviations were recorded. The total 93 participants were included in ITT analyses."

16-ii) Primary analysis should be intent-to-treat

Primary analysis should be intent-to-treat, secondary analyses could include comparing only "users", with the appropriate caveats that this is no longer a randomized sample (see 18-i).

	1	2	3	4	5	
subitem not at all important	○	○	○	○	●	essential

清除所选内容

Does your paper address subitem 16-ii?

Copy and paste relevant sections from the manuscript (include quotes in quotation marks "like this" to indicate direct quotes from your manuscript), or elaborate on this item by providing additional information not in the ms, or briefly explain why the item is not applicable/relevant for your study

"The specific research process is shown in Figure 2. 15 of 115 participants assessed for eligibility were excluded. Of these, 7 subsequently withdrew consent, and the remaining 93 participants were randomized to the intervention group (TAU + Echo App V2.0, n = 47) or the control group (TAU only, n = 46). 13 participants and 29 participants dropped out at T2 (dropout rate:14%) and T3(dropout rate:31%) respectively. With regard to intervention fidelity, 39 of 47 participants in the intervention group (83.0%) completed all 10 sessions; the mean number of sessions completed was 9.2 (SD = 1.3), with a mean total engagement time of approximately 338 minutes per participant. 6 participants completed fewer than 8 sessions, most commonly due to early discharge (n=4) or device technical issues (n=2). No major protocol deviations were recorded. The total 93 participants were included in ITT analyses."

17a) For each primary and secondary outcome, results for each group, and the estimated effect size and its precision (such as 95% confidence interval)

Does your paper address CONSORT subitem 17a? *

Copy and paste relevant sections from the manuscript (include quotes in quotation marks "like this" to indicate direct quotes from your manuscript), or elaborate on this item by providing additional information not in the ms, or briefly explain why the item is not applicable/relevant for your study

"Both groups showed an increase in craving after discharge. Figure 3 illustrated that the time effect on the craving scores was significant between T0 and T2 (β= 5.349, 95% CI: [2.732, 7.967], p < 0.001, Cohen's d =1.885) and T3 (β = 5.002, 95% CI: [2.518, 7.486], p < 0.001, Cohen's d =1.763). However, the effect of T1 was not significant (β = -0.546, 95% CI: [-3.167, 2.074], p = 0.682, Cohen's d = -0.193). The effect of the intervention was not significant (β = 0.225, 95% CI: [-0.973, 1.423], p = 0.713, Cohen's d = 0.079). The interaction effect between time and group was significant at T2 (β = -1.805, 95% CI: [-3.445, -0.164], p = 0.033, Cohen's d = -0.636) and T3 (β = -1.965, 95% CI: [-3.606, -0.324], p = 0.020, Cohen's d = -0.693). Specifically, the intervention group showed significantly attenuated craving increase at week 8 and week 16 .Table S3 provides the absolute estimated levels at each time point for direct comparison."

17a-i) Presentation of process outcomes such as metrics of use and intensity of use

In addition to primary/secondary (clinical) outcomes, the presentation of process outcomes such as metrics of use and intensity of use (dose, exposure) and their operational definitions is critical. This does not only refer to metrics of attrition (13-b) (often a binary variable), but also to more continuous exposure metrics such as "average session length". These must be accompanied by a technical description how a metric like a "session" is defined (e.g., timeout after idle time) [1] (report under item 6a).

	1	2	3	4	5	
subitem not at all important	○	○	○	○	◉	essential

清除所选内容

Does your paper address subitem 17a-i?

Copy and paste relevant sections from the manuscript (include quotes in quotation marks "like this" to indicate direct quotes from your manuscript), or elaborate on this item by providing additional information not in the ms, or briefly explain why the item is not applicable/relevant for your study

"The specific research process is shown in Figure 2. 15 of 115 participants assessed for eligibility were excluded. Of these, 7 subsequently withdrew consent, and the remaining 93 participants were randomized to the intervention group (TAU + Echo App V2.0, n = 47) or the control group (TAU only, n = 46). 13 participants and 29 participants dropped out at T2 (dropout rate:14%) and T3(dropout rate:31%) respectively. With regard to intervention fidelity, 39 of 47 participants in the intervention group (83.0%) completed all 10 sessions; the mean number of sessions completed was 9.2 (SD = 1.3), with a mean total engagement time of approximately 338 minutes per participant. 6 participants completed fewer than 8 sessions, most commonly due to early discharge (n=4) or device technical issues (n=2). No major protocol deviations were recorded. The total 93 participants were included in ITT analyses."

17b) For binary outcomes, presentation of both absolute and relative effect sizes is recommended

Does your paper address CONSORT subitem 17b? *

Copy and paste relevant sections from the manuscript (include quotes in quotation marks "like this" to indicate direct quotes from your manuscript), or elaborate on this item by providing additional information not in the ms, or briefly explain why the item is not applicable/relevant for your study

"Relapse rate at T2 and T3 were 86.02% and 61.29 %, respectively. At T2, 55 participants relapsed while 38 achieved remissions. In the intervention group, 23 participants relapsed and 24 remitted (relapse rate:48.9%). For the control group, 32 participants relapsed and 14 remitted (relapse rate:69.6%).
The chi-square analysis found significant difference between two groups (Figure 3, $\chi 2=4.094$, $p=0.043$). At T3, 68 relapsed and 25 remitted. In the intervention group, 34 participants relapsed and 13 remitted (relapse rate:72.3%). For the control group, 34 participants relapsed and 12 remitted (relapse rate:73.9%). The chi-square analysis indicated no significant difference (Figure 3c, $\chi 2=0.029$, $p=0.864$)."

18) Results of any other analyses performed, including subgroup analyses and adjusted analyses, distinguishing pre-specified from exploratory

Does your paper address CONSORT subitem 18? *

Copy and paste relevant sections from the manuscript (include quotes in quotation marks "like this" to indicate direct quotes from your manuscript), or elaborate on this item by providing additional information not in the ms, or briefly explain why the item is not applicable/relevant for your study

"For the difference between VAS scores at T2 and T0 (Figure 5), the overall model was statistically significant (F (2, 90) = 4.841, p = 0.010), with an R^2 value of 0.097. After adjusting for the predictors, the adjusted R^2 was 0.077. The intercept of the model was 3.40 (95% CI: [2.07, 4.72], p < 0.001). There was a significant negative effect of intervention on the VAS change (β = -2.133, 95% CI: [-4.032, -0.235], p = 0.028). Additionally, higher PSS scores were significantly associated with a greater increase in craving change (β = 0.146, 95% CI: [0.028, 0.263], p = 0.016). Variance inflation factors (VIF = 1.02) indicated no multicollinearity between predictors. The Breusch-Pagan test (BP = 3.82, p = .148) confirmed homoscedasticity of residuals, and the Shapiro-Wilk test (W = 0.979, p = .137) verified that residuals were normally distributed. Ten-fold cross-validation yielded an R^2 of 0.138, exceeding the in-sample R^2 of 0.097, indicating that the model did not overfit the data.

For the difference between craving score at T3 and T0, the overall model (Figure 5) was statistically significant (F (2, 90) = 4.677, p = 0.012, R^2= 0.094). After adjusting for the predictors, the adjusted R^2 was 0.074. There was a significant negative effect of intervention on the craving change (β= -2.243, 95% CI: [-4.067, -0.419], p = 0.017). Higher PSS scores were significantly associated with a greater increase in craving change (β = 0.123, 95% CI: [0.010, 0.236], p = 0.033). Variance inflation factors (VIF = 1.02) indicated no multicollinearity between predictors. Ten-fold cross-validation yielded an R^2 of 0.162, exceeding the in-sample R^2 of 0.094, demonstrating satisfactory generalizability. Although the Shapiro-Wilk test indicated mild non-normality of residuals (W = 0.961, p = .007), bootstrap confidence intervals (1,000 resamples) were additionally computed to ensure inferential robustness. Bootstrap results were consistent with those obtained from parametric testing, confirming that the regression coefficients and their significance were not meaningfully affected by the departure from normality.

###Figure 5 ###

The logistic regression analysis used relapse at T2 as the dependent variable (Figure 6). The intervention group had significantly lower odds of relapse compared to the control group (β = 1.019, OR = 2.770, 95% CI: [1.111, 6.909], p = 0.029). Additionally, abstinence frequency was positively associated with the odds of remission (β = 0.273, OR = 1.314, 95% CI: [1.062, 1.628], p = 0.011).

###Figure6###

"

18-i) Subgroup analysis of comparing only users

A subgroup analysis of comparing only users is not uncommon in ehealth trials, but if done, it must be stressed that this is a self-selected sample and no longer an unbiased sample from a randomized trial (see 16-iii).

	1	2	3	4	5	
subitem not at all important	○	○	○	○	●	essential

清除所选内容

Does your paper address subitem 18-i?

Copy and paste relevant sections from the manuscript (include quotes in quotation marks "like this" to indicate direct quotes from your manuscript), or elaborate on this item by providing additional information not in the ms, or briefly explain why the item is not applicable/relevant for your study

"The specific research process is shown in Figure 2. 15 of 115 participants assessed for eligibility were excluded. Of these, 7 subsequently withdrew consent, and the remaining 93 participants were randomized to the intervention group (TAU + Echo App V2.0, n = 47) or the control group (TAU only, n = 46). 13 participants and 29 participants dropped out at T2 (dropout rate:14%) and T3(dropout rate:31%) respectively. With regard to intervention fidelity, 39 of 47 participants in the intervention group (83.0%) completed all 10 sessions; the mean number of sessions completed was 9.2 (SD = 1.3), with a mean total engagement time of approximately 338 minutes per participant. 6 participants completed fewer than 8 sessions, most commonly due to early discharge (n=4) or device technical issues (n=2). No major protocol deviations were recorded. The total 93 participants were included in ITT analyses."

19) All important harms or unintended effects in each group

(for specific guidance see CONSORT for harms)

Does your paper address CONSORT subitem 19? *

Copy and paste relevant sections from the manuscript (include quotes in quotation marks "like this" to indicate direct quotes from your manuscript), or elaborate on this item by providing additional information not in the ms, or briefly explain why the item is not applicable/relevant for your study

"Adverse events and participant safety were monitored throughout the study. No serious adverse events related to the intervention were recorded. Participants showing signs of clinical deterioration were referred to the clinical team for evaluation. No participants were withdrawn from the study due to intervention-related harm. Digital interactions were reviewed by the clinical supervisor weekly to identify any unexpected distress."

19-i) Include privacy breaches, technical problems

Include privacy breaches, technical problems. This does not only include physical "harm" to participants, but also incidents such as perceived or real privacy breaches [1], technical problems, and other unexpected/unintended incidents. "Unintended effects" also includes unintended positive effects [2].

	1	2	3	4	5	
subitem not at all important	○	○	○	○	◉	essential

清除所选内容

Does your paper address subitem 19-i?

Copy and paste relevant sections from the manuscript (include quotes in quotation marks "like this" to indicate direct quotes from your manuscript), or elaborate on this item by providing additional information not in the ms, or briefly explain why the item is not applicable/relevant for your study

"Adverse events and participant safety were monitored throughout the study. No serious adverse events related to the intervention were recorded. Participants showing signs of clinical deterioration were referred to the clinical team for evaluation. No participants were withdrawn from the study due to intervention-related harm. Digital interactions were reviewed by the clinical supervisor weekly to identify any unexpected distress."

19-ii) Include qualitative feedback from participants or observations from staff/researchers

Include qualitative feedback from participants or observations from staff/researchers, if available, on strengths and shortcomings of the application, especially if they point to unintended/unexpected effects or uses. This includes (if available) reasons for why people did or did not use the application as intended by the developers.

	1	2	3	4	5	
subitem not at all important	◉	○	○	○	○	essential

清除所选内容

Does your paper address subitem 19-ii?

Copy and paste relevant sections from the manuscript (include quotes in quotation marks "like this" to indicate direct quotes from your manuscript), or elaborate on this item by providing additional information not in the ms, or briefly explain why the item is not applicable/relevant for your study

您的回答

DISCUSSION

22) Interpretation consistent with results, balancing benefits and harms, and considering other relevant evidence

NPT: In addition, take into account the choice of the comparator, lack of or partial blinding, and unequal expertise of care providers or centers in each group

22-i) Restate study questions and summarize the answers suggested by the data, starting with primary outcomes and process outcomes (use)

Restate study questions and summarize the answers suggested by the data, starting with primary outcomes and process outcomes (use).

	1	2	3	4	5	
subitem not at all important	○	○	○	○	◉	essential

清除所选内容

Does your paper address subitem 22-i? *

Copy and paste relevant sections from the manuscript (include quotes in quotation marks "like this" to indicate direct quotes from your manuscript), or elaborate on this item by providing additional information not in the ms, or briefly explain why the item is not applicable/relevant for your study

"This study is, to our knowledge, among the first RCTs with mid-term follow-up (up to 16 weeks post-discharge) to test the effectiveness of VA-based digital intervention in AUD during hospitalization. The intervention group demonstrated a significantly attenuated increase in craving at week 8 and week 16 post-discharge and a lower relapse rate at the 8-week follow-up compared to TAU alone, suggesting short-term suppression of craving by the intervention. Additionally, the intervention group experienced greater improvements in sleep quality. However, the between-group difference in relapse rates was no longer significant at the 16-week mid-term follow-up. Linear regression analysis also suggest that stress can effectively predict changes in craving at two follow-ups post discharge. The logistic regression showed that both the intervention and abstinence frequency can predict relapse."

22-ii) Highlight unanswered new questions, suggest future research

Highlight unanswered new questions, suggest future research.

	1	2	3	4	5	
subitem not at all important	○	○	○	○	◉	essential

清除所选内容

Does your paper address subitem 22-ii?

Copy and paste relevant sections from the manuscript (include quotes in quotation marks "like this" to indicate direct quotes from your manuscript), or elaborate on this item by providing additional information not in the ms, or briefly explain why the item is not applicable/relevant for your study

"VA-based digital interventions have great potential in reducing alcohol among general population. VA could achieve better user experience through empathy, nonverbal relational behaviors, and disclosure of personal information[74]. Digital interventions based on VA have showed effectiveness in sleep regularity, depression prevention, distress and anxiety reduction and adherence improvement [19, 75-78]. "

20) Trial limitations, addressing sources of potential bias, imprecision, and, if relevant, multiplicity of analyses

20-i) Typical limitations in ehealth trials

Typical limitations in ehealth trials: Participants in ehealth trials are rarely blinded. Ehealth trials often look at a multiplicity of outcomes, increasing risk for a Type I error. Discuss biases due to non-use of the intervention/usability issues, biases through informed consent procedures, unexpected events.

	1	2	3	4	5	
subitem not at all important	○	○	○	○	●	essential

清除所选内容

Does your paper address subitem 20-i? *

Copy and paste relevant sections from the manuscript (include quotes in quotation marks "like this" to indicate direct quotes from your manuscript), or elaborate on this item by providing additional information not in the ms, or briefly explain why the item is not applicable/relevant for your study

"Further studies should validate the potential of Echo App V2.0 to provide self-management support for patients post discharge. Specifically, future work should assess the feasibility of deploying Echo App V2.0 on patients' own devices following discharge, evaluate the acceptability of a post-discharge "booster" session schedule, and examine whether continued app engagement mediates longer-term relapse outcomes. The post-discharge phase represents a critical period in AUD recovery during which patients face heightened exposure to environmental triggers, stress, and social pressure to drink. An app-delivered self-management tool that extends the therapeutic frameworks already introduced during hospitalization, such as CBT-based coping strategies and mindfulness techniques could provide timely and accessible support precisely when it is most needed. Besides, more personalized treatment plans can be further implemented in the future. "

21) Generalisability (external validity, applicability) of the trial findings

NPT: External validity of the trial findings according to the intervention, comparators, patients, and care providers or centers involved in the trial

21-i) Generalizability to other populations

Generalizability to other populations: In particular, discuss generalizability to a general Internet population, outside of a RCT setting, and general patient population, including applicability of the study results for other organizations

	1	2	3	4	5	
subitem not at all important	○	○	○	○	●	essential

清除所选内容

Does your paper address subitem 21-i?

Copy and paste relevant sections from the manuscript (include quotes in quotation marks "like this" to indicate direct quotes from your manuscript), or elaborate on this item by providing additional information not in the ms, or briefly explain why the item is not applicable/relevant for your study

"Third, all participants were recruited from a single inpatient facility, and findings may not extend to outpatient clinics, community settings, or primary care, where patients typically present with less severe AUD and different environmental triggers. The study's age restriction precludes generalization to older adults or adolescents, who may differ in technology engagement and treatment needs. Multicenter, multi-setting trials enrolling more diverse samples are needed before broad clinical implementation. "

21-ii) Discuss if there were elements in the RCT that would be different in a routine application setting

Discuss if there were elements in the RCT that would be different in a routine application setting (e.g., prompts/reminders, more human involvement, training sessions or other co-interventions) and what impact the omission of these elements could have on use, adoption, or outcomes if the intervention is applied outside of a RCT setting.

	1	2	3	4	5	
subitem not at all important	○	○	○	○	◉	essential

清除所选内容

Does your paper address subitem 21-ii?

Copy and paste relevant sections from the manuscript (include quotes in quotation marks "like this" to indicate direct quotes from your manuscript), or elaborate on this item by providing additional information not in the ms, or briefly explain why the item is not applicable/relevant for your study

"Third, all participants were recruited from a single inpatient facility, and findings may not extend to outpatient clinics, community settings, or primary care, where patients typically present with less severe AUD and different environmental triggers. The study's age restriction precludes generalization to older adults or adolescents, who may differ in technology engagement and treatment needs. Multicenter, multi-setting trials enrolling more diverse samples are needed before broad clinical implementation. "

OTHER INFORMATION

23) Registration number and name of trial registry

Does your paper address CONSORT subitem 23? *

Copy and paste relevant sections from the manuscript (include quotes in quotation marks "like this" to indicate direct quotes from your manuscript), or elaborate on this item by providing additional information not in the ms, or briefly explain why the item is not applicable/relevant for your study

"(Trial registration: ClinicalTrials.gov Identifier: NCT05675553)"

24) Where the full trial protocol can be accessed, if available

Does your paper address CONSORT subitem 24? *

Cite a Multimedia Appendix, other reference, or copy and paste relevant sections from the manuscript (include quotes in quotation marks "like this" to indicate direct quotes from your manuscript), or elaborate on this item by providing additional information not in the ms, or briefly explain why the item is not applicable/relevant for your study

Protocol has been uploaded as well.

25) Sources of funding and other support (such as supply of drugs), role of funders

Does your paper address CONSORT subitem 25? *

Copy and paste relevant sections from the manuscript (include quotes in quotation marks "like this" to indicate direct quotes from your manuscript), or elaborate on this item by providing additional information not in the ms, or briefly explain why the item is not applicable/relevant for your study

"Funding: This research protocol was funded by STI2030-Major Projects (2021ZD0202105), the National Natural Science Foundation of China (82130041, 82201650，82171483), Shanghai "Rising Stars of Medical Talents" Youth Development Program (SHWSRS(2025)_071), Shanghai Rising-star Cultivation Program (22YF1439200), Shanghai Clinical Research Center for Mental Health (19MC1911100), Xuhui District Technology Cooperation Project (23XHYD-09) and Fuyang Health and Wellness Scientific Research Project(FY2023-04)."

X27) Conflicts of Interest (not a CONSORT item)

X27-i) State the relation of the study team towards the system being evaluated

In addition to the usual declaration of interests (financial or otherwise), also state the relation of the study team towards the system being evaluated, i.e., state if the authors/evaluators are distinct from or identical with the developers/sponsors of the intervention.

	1	2	3	4	5	
subitem not at all important	○	○	○	○	◉	essential

清除所选内容

Does your paper address subitem X27-i?

Copy and paste relevant sections from the manuscript (include quotes in quotation marks "like this" to indicate direct quotes from your manuscript), or elaborate on this item by providing additional information not in the ms, or briefly explain why the item is not applicable/relevant for your study

"Competing Interests: All authors declare no potential conflicts of interest, including specific financial interests and relationships and affiliations relevant to the subject of this manuscript."

About the CONSORT EHEALTH checklist

As a result of using this checklist, did you make changes in your manuscript? *

- ○ yes, major changes
- ◉ yes, minor changes
- ○ no

What were the most important changes you made as a result of using this checklist?

您的回答

How much time did you spend on going through the checklist INCLUDING making changes in your manuscript *

I spend 3 weeks on going through the checklist INCLUDING making changes in your manuscript

As a result of using this checklist, do you think your manuscript has improved? *

- ◉ yes
- ○ no
- ○ 其他:

Would you like to become involved in the CONSORT EHEALTH group?

This would involve for example becoming involved in participating in a workshop and writing an "Explanation and Elaboration" document

- (x) yes
- ( ) no
- ( ) 其他:

清除所选内容

Any other comments or questions on CONSORT EHEALTH

您的回答

STOP - Save this form as PDF before you click submit

To generate a record that you filled in this form, we recommend to generate a PDF of this page (on a Mac, simply select "print" and then select "print as PDF") before you submit it.

When you submit your (revised) paper to JMIR, please upload the PDF as supplementary file.

Don't worry if some text in the textboxes is cut off, as we still have the complete information in our database. Thank you!

Final step: Click submit !

Click submit so we have your answers in our database!

提交 清除表单内容

切勿通过 Google 表单提交密码。

此内容不是由 Google 所创建，Google 不对其作任何担保。 - 联系表单所有者 - 服务条款 - 隐私权政策

此表单看起来很可疑吗？ 报告

Google 表单
