# Supplementary material for: Efficacy of A Virtual Agent–Based Digital Intervention (Echo App V2.0) for Patients With Alcohol Use Disorder: Randomized Controlled Trial
Source: J Med Internet Res. 2026 Sep 17;28:e91065. doi: 10.2196/91065 (PMC13585020; doi:10.2196/91065)
Supplement: Supplementary file 2 [file jmir-v28-e91065-s001.docx]

| Table S1. Details of virtual agent-assisted intelligent rehabilitation treatment sessions | | | |
| --- | --- | --- | --- |
| Session | Theme | Details of intervention based on CBT | Corresponding dimensions |
| 1 | Enhancing motivation for abstinence | This session introduces the dangers of alcohol, including physical harm and psychological dependence. This session also introduces the personal experiences of people who have successfully withdrawn to encourage participants. Analytical tools such as pros and cons tables are also used. | Motivation for treatment |
| 2 | Improving participants’ understanding of alcohol cravings and triggers | This module introduces the triggers of alcohol addiction, guides participants to recall drinking situations and complete the drinking situation table. The physiological mechanism of craving and coping skills (distraction, relaxation training) are explained. | Impulsivity |
| 3 | High-risk situation identification and response | This session guides participants to identify high-risk situations of alcohol use. Participants need to complete the external triggering factor table and risk level evaluation table. Coping skills based on high-risk situations- sober breathing space and alternative behavior are given. | Coping style |
| 4 | Dealing with Negative Cognition | This session explains the ABC theory to the patient, introduce the connection between events, thoughts, emotions, and on this basis, introduce the thought record change table and common types of cognitive errors that affect thinking. | Anxiety; depression |
| 5 | Understanding emotions | This session introduces the concept of emotions management, and guides participants to understand their emotions. Based on understanding emotions, techniques for managing emotions are taught. | Anxiety; depression |
| 6 | Stress management | This session introduces knowledge about stress, including the sources of stress and how stress is characterized in different stages. Patients are informed how to perceive stressful situations, and possible associations between substance use and stress. Techniques such as stressful events sequencing, resilience improvement, and mediation relaxation are introduced to help participants cope with stress. | Stress |
| 7 | Understanding family conflicts | This session focuses on family conflicts and provides a new perspective on understanding family issues. Defense mechanisms are introduced to further explain the relationship between family members. | Anxiety; depression |
| 8 | Strengthening the participants ' self-control ability to avoid relapse | This session firstly introduces internal and external relapse rationalization excuses, and has participants complete their own analysis of potential excuses. Then it introduces the key factors that affect relapse such as "rational self", "addicted self" and "seemingly irrelevant decisions", and finally guides participants to learn the skills of coping with relapse - making early warning cards, weighing the advantages and disadvantages, and relaxation mediation. | Compulsion |
| 9 | Mindfulness Practice | This session systematically introduces the background and history of mindfulness techniques. Through the practice of sitting mediation and mountain mediation, participants develop understanding of mindfulness. | Sleep quality; |
| 10 | Promoting participants’ self-awareness of happiness and well-being | This session aims to promote participants ' self-awareness and introduces discussion about well-being - recognizing different types of well-being, techniques and methods on how to improve well-being and the psychology of Mandala. | Anxiety; depression |

| Table S2. Secondary outcome measures(detailed) | | | | | | | | |
| --- | --- | --- | --- | --- | --- | --- | --- | --- |
| **Measurements** | Intervention group (Mean,SD) | Control group (Mean,SD) | Group | | Time | | Group *Time | |
|  |  |  | β[95%CI] | *p* | β[95%CI] | *p* | β[95%CI] | *p* |
| **PHQ-9** |  |  |  |  |  |  |  |  |
| T0 | 8.64 (6.50) | 9.85 (7.04) | -1.197[-4.213,1.819] | 0.430 | 0.870[-1.282,3.022] | 0.424 | -2.657[-5.725, 0.412] | 0.084 |
| T1 | 6.85 (6.69) | 10.7 (8.57) |  |  |  |  |  |  |
| **GAD-7** |  |  |  |  |  |  |  |  |
| T0 | 6.19 (5.22) | 6.74 (5.16) | -0.471[-2.848, 1.906] | 0.697 | 1.196[-0.475,2.868] | 0.152 | -1.877[-4.171, 0.417] | 0.110 |
| T1 | 5.51 (6.19) | 7.93 (6.44) |  |  |  |  |  |  |
| **PSS** |  |  |  |  |  |  |  |  |
| T0 | 14.60 (6.43) | 17.00 (6.13) | -2.424[-5.301, 0.453] | 0.090 | -2.913[-5.267,-0.559] | 0.016 | 2.253[-1.091, 5.597] | 0.182 |
| T1 | 13.90 (7.70) | 14.10 (6.83) |  |  |  |  |  |  |
| **SCORATES** |  |  |  |  |  |  |  |  |
| T0 | 66.8 (17.7) | 60.4 (18.5) | 6.550[-1.666, 14.766] | 0.117 | 0.065 [-5.587, 5.718] | 0.982 | −0.661[-8.598, 7.275] | 0.870 |
| T1 | 66.2 (22.1) | 60.5 (20.8) |  |  |  |  |  |  |
| **PSQI** |  |  |  |  |  |  |  |  |
| T0 | 7.53 (4.61) | 6.22 (3.44) | 1.330[-0.150, 2.810] | 0.097 | 0.304[-0.973,1.582] | 0.640 | -3.283[-5.080, -1.485] | *p* <0.001 |
| T1 | 4.55 (3.13) | 6.52 (3.89) |  |  |  |  |  |  |

Footnote: T0: baseline; T1: week 4

| Table S3.Estimated marginal mean of two groups at timepoints | | | | | |
| --- | --- | --- | --- | --- | --- |
| Time | Control EMM | Intervention EMM | Difference | t | *p* |
|  | *[95% CI]* | *[95% CI]* | *[95% CI]* |  |  |
| T0 | 2.04 [0.11, 3.96] | 2.26 [1.40, 3.12] | −0.22 [−1.44, 0.99] | −0.369 | 0.713 |
| T1 | 1.49 [−0.43, 3.41] | 1.87 [1.01, 2.73] | −0.38 [−1.59, 0.83] | −0.623 | 0.535 |
| T2 | 7.38 [5.46, 9.31] | 5.80 [4.94, 6.67] | 1.58 [0.37, 2.79] | 2.589 | 0.011 |
| T3 | 7.04 [5.12, 8.96] | 5.30 [4.44, 6.16] | 1.74 [0.53, 2.95] | 2.851 | 0.005 |
| *EMM = estimated marginal mean, derived from the linear mixed-effects model (LME). Difference direction: Control − Intervention; a positive value indicates higher scores in the control group.  T0: Baseline; T1: Week 4 follow-up; T2: One month follow-up; T3: Three-month follow-up | | | | | |

| Table S4.GLMM and Multiple Imputation Estimates of Group-by-Time Interaction Effects on VAS | | | | | | | | | |
| --- | --- | --- | --- | --- | --- | --- | --- | --- | --- |
| Variable | GLMM | | | | | MI | | | |
|  | β | 95% CI | t | *p* | β | | 95% CI | t | *p* |
| Group (baseline) | 0.225 | [−0.971, 1.421] | 0.369 | 0.713 | 0.349 | | [−0.926, 1.624] | 0.538 | 0.591 |
| Time T1 | −0.546 | [−3.162, 2.071] | −0.409 | 0.683 | −0.342 | | [−3.054, 2.371] | −0.248 | 0.805 |
| Time T2 | 5.349 | [2.732, 7.965] | 4.008 | < 0.001 | 6.258 | | [3.441, 9.075] | 4.371 | < 0.001 |
| Time T3 | 5.002 | [2.385, 7.618] | 3.748 | < 0.001 | 7.734 | | [4.886, 10.583] | 5.345 | < 0.001 |
| Group × T1 | 0.155 | [−1.494, 1.804] | 0.184 | 0.854 | 0.053 | | [−1.652, 1.757] | 0.061 | 0.952 |
| Group × T2 | −1.805 | [−3.454, −0.156] | −2.146 | 0.033 | −2.453 | | [−4.245, −0.662] | −2.696 | 0.007 |
| Group × T3 | −1.965 | [−3.614, −0.316] | −2.336 | 0.02 | −3.549 | | [−5.384, −1.713] | −3.809 | < 0.001 |
| *GLMM:Generalized Linear Mixed Model;MI:Multiple Imputation  T0: Baseline; T1: Week 4 follow-up; T2: One-month follow-up; T3: Three month follow-up | | | | | | | | | |

| Table S5.Linear Mixed-Effects Model Results for Coping Styles and Impulsivity | | | | | |
| --- | --- | --- | --- | --- | --- |
| **Outcome** | **Effect** | **β** | **95% CI** | ***p*** | **Cohen’s d** |
| SCSQ Positive Coping | Group | 1.152 | [-3.176, 5.479] | 0.603 | 0.108 |
| SCSQ Positive Coping | Time | 4.196 | [-0.155, 8.546] | 0.06 | 0.394 |
| SCSQ Positive Coping | Group × Time | -2.174 | [-8.295, 3.946] | 0.487 | -0.204 |
| SCSQ Negative Coping | Group | -0.966 | [-3.917, 1.986] | 0.522 | -0.14 |
| SCSQ Negative Coping | Time | -1.174 | [-3.990, 1.642] | 0.416 | -0.17 |
| SCSQ Negative Coping | Group × Time | 3.365 | [-0.596, 7.326] | 0.099 | 0.488 |
| BIS | Group | -12.21 | [-18.370, -6.051] | <0.001 | -0.824 |
| BIS | Time | -9.022 | [-15.078, -2.965] | 0.004 | -0.609 |
| BIS | Group × Time | 12.979 | [4.460, 21.499] | 0.004 | 0.876 |
| *.SCSQ, Simplified Coping Style Questionnaire; BIS, Barratt Impulsiveness Scale; CI, confidence interval. Group compares Treatment with Control. Time compares Post with Pre. Group × Time represents the interaction effect between group and time. β = fixed-effect estimate; d = Cohen’s d. | | | | | |

| Table S6. Mean and Interquartile Range of Outcome Measures at Each Time Point | | | | | |
| --- | --- | --- | --- | --- | --- |
|  |  | Control | | Intervention | |
|  |  | Mean | IQR | Mean | IQR |
| VAS | T0 | 2.26 | 4（0–4） | 2.49 | 5（0–5） |
|  | T1 | 1.87 | 2（0–2） | 2.24 | 3.5（0–3.5） |
|  | T2 | 5.80 | 5.5（3.5–9） | 4.21 | 5（1–6） |
|  | T3 | 5.30 | 3.5（4.25–7.75） | 3.55 | 4.5（0–4.5） |
| PHQ-9 | T0 | 9.85 | 10（4–14） | 8.64 | 8.5（4.5–13） |
|  | T1 | 10.72 | 15（3–18） | 6.85 | 7.5（2–9.5） |
| GAD-7 | T0 | 6.74 | 7（2–9） | 6.19 | 5.5（3–8.5） |
|  | T1 | 7.93 | 12（2–14） | 5.51 | 10（0–10） |
| PSQI | T0 | 6.22 | 4.75（4–8.75） | 7.53 | 9（3–12） |
|  | T1 | 6.52 | 6（3–9） | 4.55 | 3.5（3–6.5） |
| PSS | T0 | 16.98 | 7.75（13–21） | 14.57 | 10.5（9.5–20） |
|  | T1 | 14.07 | 8.75（10.25–19） | 13.91 | 9（10–19） |
| SCORATES | T0 | 60.41 | 20.5（52.25–72.75） | 66.83 | 19.5（57–76.5） |
|  | T1 | 60.48 | 26.5（47–73.5） | 66.23 | 30（51.5–81.5） |
| BIS | T0 | 74.90 | 35.80（60.20–96.00） | 62.70 | 10.50（60.50–71.00） |
|  | T1 | 65.90 | 11.80（60.00–71.80） | 66.60 | 12.50（60.00–72.50） |
| SCSQ（Positive Coping） | T0 | 14.90 | 19.50（5.25–24.80） | 16.00 | 17.00（6.50–23.50） |
|  | T1 | 19.10 | 18.75（9.25–28.00） | 18.00 | 16.00（11.00–27.00） |
| SCSQ(Negative Coping) | T0 | 12.40 | 15.00（4.00–19.00） | 11.40 | 13.00（5.50–18.50） |
|  | T1 | 11.20 | 10.75（5.25–16.00） | 13.60 | 9.50（9.50–19.00） |
| Alcohol use(Relapse/Remit) | T2 | 32/14 | / | 23/24 | / |
|  | T3 | 34/12 | / | 34/13 | / |
| *Intervention Group(Echo App V2.0 + TAU, n=47); Control Group[(TAU, n=46) PHQ-9: Patient Health Questionnaire-9; GAD-7: Generalized Anxiety Disorder-7; PSS: Perceived Stress Scale; SCORATES: Stages of Change Readiness and Treatment Eagerness Scale; PSQI: Pittsburgh Sleep Quality Index;BIS: Barratt Impulsiveness Scale; SCSQ, Simplified Coping Style Questionaire T0:baseline, T1:week 4 , T2: one month after discharge, T3:three months after discharge | | | | | |
|  |  |  |  |  |  |
